# Disparities in Coronavirus Disease 2019 Clinical Outcomes and Vaccination Coverage Among Migrants With Human Immunodeficiency Virus in the PISCIS Cohort: A Population-Based Propensity Score–Matched Analysis

**DOI:** 10.1093/ofid/ofad693

**Published:** 2024-01-05

**Authors:** Daniel K Nomah, Yesika Díaz, Andreu Bruguera, Sergio Moreno-Fornés, Jordi Aceiton, Juliana Reyes-Urueña, Josep M Llibre, Vicenç Falcó, Arkaitz Imaz, Francisco Javier Fanjul, Joaquim Peraire, Elisabet Deig, Pere Domingo, Alexy Inciarte, Jordi Casabona, José M Miró

**Affiliations:** Department de Salut, Centre Estudis Epidemiològics sobre les Infeccions de Transmissió Sexual i Sida de Catalunya, Generalitat de Catalunya, Badalona, Spain; Institut d’Investigació Germans Trias i Pujol, Barcelona, Spain; Department de Salut, Centre Estudis Epidemiològics sobre les Infeccions de Transmissió Sexual i Sida de Catalunya, Generalitat de Catalunya, Badalona, Spain; Institut d’Investigació Germans Trias i Pujol, Barcelona, Spain; CIBER Epidemiologia y Salud Pública, Barcelona, Spain; Department de Salut, Centre Estudis Epidemiològics sobre les Infeccions de Transmissió Sexual i Sida de Catalunya, Generalitat de Catalunya, Badalona, Spain; Institut d’Investigació Germans Trias i Pujol, Barcelona, Spain; CIBER Epidemiologia y Salud Pública, Barcelona, Spain; Departament de Pediatria, d’Obstetrícia i Ginecologia i de Medicina Preventiva i de Salut Publica, Universitat Autònoma de Barcelona, Bellaterra, Spain; Department de Salut, Centre Estudis Epidemiològics sobre les Infeccions de Transmissió Sexual i Sida de Catalunya, Generalitat de Catalunya, Badalona, Spain; Institut d’Investigació Germans Trias i Pujol, Barcelona, Spain; CIBER Epidemiologia y Salud Pública, Barcelona, Spain; Department de Salut, Centre Estudis Epidemiològics sobre les Infeccions de Transmissió Sexual i Sida de Catalunya, Generalitat de Catalunya, Badalona, Spain; Institut d’Investigació Germans Trias i Pujol, Barcelona, Spain; Department de Salut, Centre Estudis Epidemiològics sobre les Infeccions de Transmissió Sexual i Sida de Catalunya, Generalitat de Catalunya, Badalona, Spain; Fight Against Infections Foundation, Hospital Universitari Germans Trias i Pujol, Badalona, Spain; Infectious Disease Division, Hospital Universitari Vall D’Hebron, Barcelona, Spain; HIV and STI Unit, Department of Infectious Diseases, Hospital Universitari de Bellvitge–IDIBELL, L’Hospitalet de Llobregat, Spain; Infectious Diseases Unit, Hospital Son Espases, Palma de Mallorca, Spain; Hospital Joan XXIII, Institut d’Investigació Sanitària Pere Virgili, Universitat Rovira i Virgili, Tarragona, Spain; CIBERINFEC, Instituto de Salud Carlos III, Madrid, Spain; Infectious Diseases Unit, Hospital General de Granollers, Granollers, Spain; Department of Infectious Diseases, HIV Infection Unit, Hospital de la Santa Creu i Sant Pau, Barcelona, Spain; Hospital Clínic-Institut d’Investigacions Biomèdiques August Pi i Sunyer, University of Barcelona, Barcelona, Spain; Department de Salut, Centre Estudis Epidemiològics sobre les Infeccions de Transmissió Sexual i Sida de Catalunya, Generalitat de Catalunya, Badalona, Spain; Institut d’Investigació Germans Trias i Pujol, Barcelona, Spain; CIBER Epidemiologia y Salud Pública, Barcelona, Spain; Departament de Pediatria, d’Obstetrícia i Ginecologia i de Medicina Preventiva i de Salut Publica, Universitat Autònoma de Barcelona, Bellaterra, Spain; CIBERINFEC, Instituto de Salud Carlos III, Madrid, Spain; Hospital Clínic-Institut d’Investigacions Biomèdiques August Pi i Sunyer, University of Barcelona, Barcelona, Spain

**Keywords:** COVID-19, HIV, migrants, SARS-CoV-2, vaccination

## Abstract

**Background:**

Coronavirus disease 2019 (COVID-19) disproportionately affects migrants and ethnic minorities, including those with human immunodeficiency virus (HIV). Comprehensive studies are needed to understand the impact and risk factors.

**Methods:**

Using data from the PISCIS cohort of people with HIV (PWH) in Catalonia, Spain, we investigated COVID-19 outcomes and vaccination coverage. Among 10 640 PWH we compared migrants and non-migrants assessing rates of severe acute respiratory syndrome coronavirus 2 (SARS-CoV-2) testing, diagnosis, and associated clinical outcomes through propensity score matching and multivariable Cox regression.

**Results:**

The cohort (mean age, 43 years; 83.5% male) included 57.4% (3053) Latin American migrants. Migrants with HIV (MWH) had fewer SARS-CoV-2 tests (67.8% vs 72.1%, *P* < .0001) but similar COVID-19 diagnoses (29.2% vs 29.4%, *P* = .847) compared to Spanish natives. Migrants had lower complete vaccination (78.9% vs 85.1%, *P* < .0001) and booster doses (63.0% vs 65.5%, *P* = .027). COVID-19 hospitalizations (8.1% vs 5.1%, *P* < .0001) and intensive care unit (ICU) admissions (2.9% vs 1.2%, *P* < .0001) were higher among migrants, with similar hospitalization duration (5.5 vs 4.0 days, *P* = .098) and mortality (3 [0.2%] vs 6 [0.4%], *P* = .510). Age ≥40 years, CD4 counts <200 cells/μL, ≥2 comorbidities, and incomplete/nonreception of the SARS-CoV-2 vaccine increased the risk of severe COVID-19 among migrants.

**Conclusions:**

MWH had lower rates of SARS-CoV-2 testing and vaccination coverage, although the rates of COVID-19 diagnosis were similar between migrants and non-migrants. Rates of COVID-19–associated hospitalizations and ICU admissions were higher among migrants in comparison with non-migrants, with similar hospitalization duration and mortality. These findings can inform policies to address disparities in future pandemic responses for MWH.

## Teaser

We compared COVID-19 outcomes between migrants and non-migrants with HIV in Catalonia, Spain. Migrants had lower testing and vaccination rates but higher hospitalization and ICU admission rates, highlighting disparities in pandemic responses for migrants with HIV.

The coronavirus disease 2019 (COVID-19) pandemic has caused immense morbidity and mortality globally, underscoring the need to safeguard the health of all, particularly vulnerable groups. As observed in previous pandemics [1], migrants and ethnic minorities are disproportionately affected by COVID-19, experiencing worse clinical outcomes compared to host populations [2, 3]. Migrants face increased risks of various health conditions due to structural factors, socioeconomic disparities, language and cultural barriers, and limited access to healthcare services [4, 5]. Public health measures implemented to curb the spread of severe acute respiratory syndrome coronavirus 2 (SARS-CoV-2), such as lockdowns and mobility restrictions, had a greater impact on migrants compared to native populations [6].

Several studies have highlighted the higher cumulative incidence and severity of COVID-19 among migrants compared to host populations. A study conducted in Spain found that migrants from sub-Saharan Africa, the Caribbean, and Latin America had a significantly higher relative risk of COVID-19 [7]. Similarly, prospective cohort studies analyzing UK Biobank data identified increased COVID-19 diagnosis rates among migrants and ethnic minority populations [8–12]. The elevated susceptibility of migrants to SARS-CoV-2 diagnosis and severe outcomes may also stem from a higher prevalence of multifaceted factors within the population, which are typically unmeasured in studies. These include mental health conditions [13], nutritional status [14], and exposure to pollutants [15], which might notably heighten their risk. These variables, often overlooked in research studies [8–12], could intricately intersect with migration experiences, potentially impacting migrants’ susceptibility to infections.

In the context of human immunodeficiency virus (HIV), studies from Spain [16], the United Kingdom (UK) [17, 18], and the United States (US) [19] have indicated that migrants with HIV (MWH) may be at a higher risk of SARS-CoV-2 acquisition and severe COVID-19. Migrants also exhibit higher rates of late HIV diagnosis and loss to care compared to native-born populations in their host countries, leading to negative impacts on HIV viral suppression and CD4 cell levels [20–23], both identified as predictors of severe COVID-19 outcomes [16, 24]. Additionally, studies on SARS-CoV-2 vaccination have reported lower vaccination rates among MWH [25].

To gain a comprehensive understanding of the impact of COVID-19 on MWH, it is crucial to ascertain whether the increased risk is due to susceptibility to SARS-CoV-2 or factors such as sociodemographic disparities and limited healthcare access. Conducting studies in settings with universal health coverage and employing effective methods to reduce confounder imbalance can enhance our understanding of the impact of COVID-19 on this population.

In this study, we leveraged a large population-based cohort of people with HIV (PWH) in Catalonia, Spain, to compare SARS-CoV-2 testing, diagnosis, hospitalization, intensive care unit (ICU) admission, hospitalization length, mortality, and vaccination coverage between MWH and a well-matched native group. Additionally, we identified the factors associated with severe COVID-19 and vaccination coverage in the migrant group. Catalonia's publicly funded healthcare system provides universal healthcare coverage to all residents, regardless of their nationality or legal status. The findings from this study can inform public health policies and improve future pandemic response strategies for this population.

## METHODS

### Study Design and Population

We conducted a retrospective cohort study using data from the Populational HIV Cohort from Catalonia and Balearic Islands (PISCIS). PISCIS is a well-established observational, prospective, multicenter, population-based cohort that has been following individuals aged ≥16 years receiving HIV care in 16 hospitals in Catalonia and 2 in the Balearic Islands since 1998. For the purpose of this study, we used only participants receiving care in Catalonia. Detailed information about the cohort can be found elsewhere [26]. To enhance the dataset, we linked the PISCIS data with information from various official administrative public health databases through the Analytical Data for Research and Innovation in Health Project of Catalonia (PADRIS) [27] to obtain data on chronic comorbidities, SARS-CoV-2 diagnosis, associated clinical outcomes, mortality, and vaccination status (Supplementary Appendix [Figure A1]). The study period spanned from 1 March 2020 to 30 April 2022. We excluded patients who were reported as deceased before 1 March 2020, as well as those who had no information regarding their country of origin. For the analysis of vaccine coverage, we further excluded subjects who were reported as deceased before 27 December 2020 and individuals who had not utilized any health services in the prior 12 months, to minimize the underestimation of vaccine uptake (Supplementary Figure 1). Characteristics of the subjects excluded are described in Supplementary Table 1. The eligible participants were divided into 2 groups based on their country of origin: migrants and non-migrants. Migrants were defined as individuals born outside of Spain. Migrants were further classified into Western European and North American, Latin American, sub-Saharan African, and other (encompassing migrants from Asia, North Africa, and the Middle East).

### Procedures

The study assessed SARS-CoV-2 testing, diagnosis, hospitalization, ICU admission, mortality, and vaccination in participants. SARS-CoV-2 diagnosis was confirmed by a nucleic acid amplification test or antigen detection from respiratory samples. Severe COVID-19 was defined as hospitalization (>24 hours), ICU admission, or death [16]. Complete vaccination was defined as 2 doses of BNT162 (Pfizer), mRNA-1273 (Moderna), or ChAdOx1-S (Oxford/AstraZeneca) vaccines, or a single dose of Janssen Ad26.COV2.S [28]. Incomplete vaccination was receiving only 1 dose of BNT162 (Pfizer), mRNA-1273 (Moderna), or ChAdOx1-S (Oxford/AstraZeneca) vaccines. Booster vaccinations referred to additional doses after completing the primary vaccination series [28].

Independent variables in the study included age, sex, and socioeconomic deprivation. Socioeconomic deprivation was classified according to the socioeconomic deprivation level index created by the Catalan government according to the basic health area of residence [29]. This index is based on 5 indicators, which are proportion of manual workers, proportion of residents with low education level, proportion with low income, rate of premature mortality, and rate of avoidable hospitalization [29]. The index produces a continuous variable of 0 to 100, with zero being the lowest level and 100 being the highest [29]. We divided our study population into a tertile, with the highest socioeconomic group being in the first tertile (least deprived) and the third tertile being the lowest (moderately/severely deprived). HIV-related variables included HIV acquisition risk groups, time since HIV diagnosis, CD4 cell count, CD4/CD8 cell ratio, plasma HIV RNA (detectable and undetectable [≤50 copies/mL]), and reception of antiretroviral therapy (ART). Chronic comorbidities were categorized into 11 groups using *International Classification of Diseases, Ninth or Tenth Revision, Clinical Modification* classifications (Supplementary Appendix [Table A1]).

### Propensity Score Matching

We used propensity score matching to adjust covariates in modeling the association between country of origin (migrants and non-migrants) and COVID-19 outcomes. We performed 2 rounds of propensity score 1:1 matching of migrants versus Spanish using nearest-neighbor algorithms with a caliper width of 0.1 of the pooled standard deviations to ensure that key baseline characteristics of the groups were adequately balanced. We matched patients by age, sex, latest CD4 cell count, latest plasma HIV RNA, and number of comorbidities. We evaluated the covariate balance before and after matching using absolute standardized mean differences (SMDs) and specified an SMD >0.1 as a relevant imbalance [30].

### Statistical Analysis

To assess the factors associated with severe COVID-19 and vaccination uptake among MWH, we used multivariable Cox proportional hazards regression models. A separate model was built for each outcome, and adjusted hazard ratios (aHRs) along with their 95% confidence intervals (95% CIs) were provided. For the models on vaccination, we defined vaccination as reception of ≥1 dose of a SARS-CoV-2 vaccine. The models were adjusted for age, sex, latest CD4 count, latest plasma HIV RNA, number of comorbidities, and reception of ART. In the model for vaccination uptake, we also included adjustment for previous SARS-CoV-2 diagnosis. Due to collinearity observed with sex in the multivariable analysis, we did not adjust for HIV acquisition risk group. The proportional assumption for all factors included in the multivariable Cox regression analysis was verified using the Schoenfeld analysis of hazard proportionality (Supplementary Appendix [Figure A2]). Statistical significance was set at *P* < .05 (2-sided). All statistical analyses were performed using R Statistical Software version 4.1.1 (R Project for Statistical Computing).

### Ethics Declaration

The PISCIS cohort study received ethical approval from the Ethics Committee of the Germans Trias i Pujol University Hospital, Badalona, Spain (EO-11-108). Patient-level information obtained from PADRIS was anonymized and deidentified prior to the analyses. This study adheres to the Strengthening the Reporting of Observational Studies in Epidemiology (STROBE) guidelines for transparent and accurate reporting of observational studies (Supplementary Appendix [Table A2]). The planning, conduct, and reporting of the study were carried out in accordance with the principles outlined in the Declaration of Helsinki, as revised in 2013.

## RESULTS

The baseline characteristics of the overall cohort before matching are shown in Table 1. A total of 18 764 PWH in the PISCIS cohort were included in the study. Median age was 45 (interquartile range [IQR], 37–53) years and 15 412 (82.1%) were male. Regarding HIV acquisition risk groups, 10 008 (53.3%) were men who have sex with men (MSM) and 2422 (12.9%) people who inject drugs (PWID); 12 169 (64.9%) had a CD4 of >500 cells/μL, 14 704 (78.4%) had an undetectable HIV viral load, 9174 (48.9%) had 2 or more chronic comorbidities, and 16 442 (87.6%) were receiving ART. A total of 7842 (41.8%) were migrants. Migrants were younger compared to non-migrants (median age, 40 [IQR, 33–48] years vs 49 [IQR, 41–56] years; SMD, 0.661) with a lower socioeconomic deprivation index (median, 35.0 [IQR, 19.3–48.5] vs 38.5 [IQR, 25.6–49.9]; SMD, 0.201), higher proportion of MSM (61.6% vs 47.4%) and fewer PWID (4.3% vs 19.1%), lower median CD4 cell count (639 [IQR, 447–854] vs 698 [IQR, 490–931] cells/μL; SMD, 0.149), higher proportion with undetectable HIV viral load (14.3 vs 9.9; SMD, 0.140), and more individuals not receiving ART (14.3% vs 11.0%). We additionally described the baseline characteristics of the overall cohort according to region of origin, which included Spain, Western Europe and North America, Latin America, sub-Saharan Africa, and others (Supplementary Table 2).

**Table 1.** Baseline Characteristics of Unmatched Migrants and Non-migrants With Human Immunodeficiency Virus in the PISCIS Cohort, 1 March 2020–30 April 2022

Characteristic	Overall PWH (N = 18 764)	Non-migrants (n = 10 922)	Migrants (n = 7842)	SMD
Sex				0.072
Male	15 412 (82.1)	8846 (81)	6566 (83.7)	
Female	3352 (17.9)	2076 (19)	1276 (16.3)	
Age, y, median (IQR)	45 (37–53)	49 (41–56)	40 (33–48)	0.661
Age category, y				0.554
16–39	6125 (32.6)	2464 (22.6)	3661 (46.7)	
40–64	11 653 (62.1)	7643 (70)	4010 (51.1)	
≥65	986 (5.3)	815 (7.5)	171 (2.2)	
Socioeconomic deprivation index	36.68 (22.93–49.42)	38.52 (25.62–49.88)	34.99 (19.25–48.45)	0.201
Socioeconomic deprivation				0.209
Least deprived	4160 (22.2)	2331 (21.3)	1829 (23.3)	
Mildly deprived	9174 (48.9)	6591 (60.3)	2583 (32.9)	
Moderately/severely deprived	5427 (28.9)	3407 (31.2)	2020 (25.8)	
Missing	424 (2.3)	230 (2.1)	194 (2.5)	
HIV acquisition risk group				0.503
PWID	2422 (12.9)	2087 (19.1)	335 (4.3)	
MSM	10 008 (53.3)	5174 (47.4)	4834 (61.6)	
Male heterosexual	2482 (13.2)	1483 (13.6)	999 (12.7)	
Female hetero/homo/bisexual	2466 (13.1)	1414 (12.9)	1052 (13.4)	
Other	538 (2.9)	289 (2.6)	249 (3.2)	
Missing	848 (4.5)	475 (4.3)	373 (4.8)	
Years since HIV diagnosis, median (IQR)	10.75 (5.13–17.73)	13.26 (7.08–20.63)	7.96 (3.25–13.02)	0.670
CD4 count (cells/μL) category				0.120
<200	743 (4)	395 (3.6)	348 (4.4)	
200–499	4027 (21.5)	2153 (19.7)	1874 (23.9)	
≥500	12 169 (64.9)	7293 (66.8)	4876 (62.2)	
Missing	1825 (9.7)	1081 (9.9)	744 (9.5)	
CD4 count (cells/μL), median (IQR)	670 (471–900)	698 (490–931)	639 (447–854)	0.149
CD4/CD8 ratio, median (IQR)	0.84 (0.56–1.19)	0.86 (0.57–1.21)	0.81 (0.54–1.16)	0.050
Plasma HIV RNA				0.140
Detectable	2200 (11.7)	1077 (9.9)	1123 (14.3)	
Undetectable	14 704 (78.4)	8709 (79.7)	5995 (76.4)	
Missing	1860 (9.9)	1136 (10.4)	724 (9.2)	
No. of comorbidities				0.640
0	5430 (28.9)	2000 (18.3)	3430 (43.7)	
1	4160 (22.2)	2331 (21.3)	1829 (23.3)	
≥2	9174 (48.9)	6591 (60.3)	2583 (32.9)	
Type of comorbidities				
Respiratory disease	3818 (20.3)	2812 (25.7)	1006 (12.8)	0.332
Cardiovascular disease	2810 (15)	2058 (18.8)	752 (9.6)	0.267
Autoimmune disease	1952 (10.4)	1365 (12.5)	587 (7.5)	0.168
Chronic kidney disease	1619 (8.6)	1155 (10.6)	464 (5.9)	0.170
Chronic liver disease	3565 (19)	2783 (25.5)	782 (10)	0.415
Neuropsychiatric conditions	9062 (48.3)	6559 (60.1)	2503 (31.9)	0.588
Diabetes (type 1 and 2)	985 (5.2)	735 (6.7)	250 (3.2)	0.164
Metabolic disease	4124 (22)	2955 (27.1)	1169 (14.9)	0.302
Cancer	1778 (9.5)	1288 (11.8)	490 (6.2)	0.194
Hypertension	3633 (19.4)	2592 (23.7)	1041 (13.3)	0.272
Obesity	1768 (9.4)	1220 (11.2)	548 (7)	0.146
Years on ART, median (IQR)	8.55 (3.95–14.21)	10.57 (5.6–16.7)	5.93 (2.6–10.79)	0.643
Receiving ART				0.099
Yes	16 442 (87.6)	9720 (89)	6722 (85.7)	
No	2322 (12.4)	1202 (11)	1120 (14.3)	

Data are presented as No. (%) unless otherwise indicated.

Abbreviations: ART, antiretroviral therapy; HIV, human immunodeficiency virus; IQR, interquartile range; MSM, men who have sex with men; PWH, people with human immunodeficiency virus; PWID, people who inject drugs; SMD, standardized mean difference.

After 1:1 propensity score matching, the matched cohort included 10 640 PWH, with 5320 belonging to each group. The median age of the matched cohort was 43 (IQR, 36–50) years, and the majority were male (8889 [83.5%]). In terms of HIV acquisition risk, 6783 (63.7%) were in the MSM group, and 443 (4.2%) were PWID. The median CD4 count was 682 (IQR, 486–900) cells/μL, with 9363 (88%) of the cohort having an undetectable HIV viral load and 9850 (92.6%) receiving ART (Table 1). The groups were comparable for sex (SMD, 0.001), age (SMD, 0.013), socioeconomic deprivation index (SMD, 0.052), HIV acquisition risk group (SMD, 0.011), CD4 cell count (SMD, 0.051), CD4/CD8 ratio (SMD, 0.045), plasma HIV RNA (SMD, 0.008), number of comorbidities (SMD, 0.028) and proportion receiving ART (SMD, 0.077). Migrants had, however, received ART in a shorter duration than non-migrants (7.5 years vs 9.0 years; SMD, 271) (Table 2).

**Table 2.** Propensity Score–Matched Baseline Characteristics of Migrants and Non-migrants With Human Immunodeficiency Virus in the PISCIS Cohort, 1 March 2020–30 April 2022

Characteristic	Total (N = 10 640)	Non-migrants (n = 5320)	Migrants^a^ (n = 5320)	SMD
Sex				0.001
Male	8889 (83.5)	4445 (83.6)	4444 (83.5)	
Female	1751 (16.5)	875 (16.4)	876 (16.5)	
Age, y, median (IQR)	43 (36–50)	43 (36–51)	43 (36–50)	0.013
Age category, y				0.007
16–39	3939 (37)	1966 (37)	1973 (37.1)	
40–64	6422 (60.4)	3217 (60.5)	3205 (60.2)	
≥65	279 (2.6)	137 (2.6)	142 (2.7)	
Socioeconomic deprivation index	35.4 (19.6–48.6)	36.1 (22.9–48.6)	35.0 (19.3–48.6)	0.052
Socioeconomic deprivation, category				0.103
Least deprived	5663 (53.2)	2733 (51.4)	2930 (55.1)	
Mildly deprived	1929 (18.1)	1064 (20.0)	865 (16.3)	
Moderately/severely deprived	2796 (26.3)	1404 (26.4)	1392 (26.2)	
Missing	252 (2.4)	119 (2.2)	133 (2.5)	
HIV acquisition risk group				0.011
PWID	443 (4.2)	226 (4.2)	217 (4.1)	
MSM	6783 (63.7)	3386 (63.6)	3397 (63.9)	
Male heterosexual	1563 (14.7)	783 (14.7)	780 (14.7)	
Female hetero/homo/bisexual	1536 (14.4)	770 (14.5)	766 (14.4)	
Other	315 (3)	155 (2.9)	160 (3)	
Years since HIV diagnosis, median (IQR)	9.7 (4.9–15.1)	10.1 (5.1–16.3)	9.3 (4.7–14.0)	0.172
CD4 count (cells per μL) category				0.056
<200	408 (3.8)	204 (3.8)	204 (3.8)	
200–499	2396 (22.5)	1136 (21.4)	1260 (23.7)	
≥500	7836 (73.6)	3980 (74.8)	3856 (72.5)	
CD4 count (cells per μL), median (IQR)	682.0 (486.0–900.0)	694.0 (498.0–910.0)	672.0 (479.0–888.3)	0.051
CD4/CD8 ratio, median (IQR)	0.89 (0.6–1.2)	0.88 (0.6–1.2)	0.85 (0.58–1.2)	0.045
Plasma HIV RNA				0.008
Detectable	1277 (12.0)	645 (12.1)	632 (11.9)	
Undetectable	9363 (88.0)	4675 (87.9)	4688 (88.1)	
No. of comorbidities				0.028
0	3334 (31.3)	1633 (30.7)	1701 (32)	
1	2923 (27.5)	1471 (27.7)	1452 (27.3)	
≥2	4383 (41.2)	2216 (41.7)	2167 (40.7)	
Type of comorbidities				
Respiratory disease	1807 (17.0)	980 (18.4)	827 (15.5)	0.077
Cardiovascular disease	1227 (11.5)	610 (11.5)	617 (11.6)	0.004
Autoimmune disease	1050 (9.9)	546 (10.3)	504 (9.5)	0.026
Chronic kidney disease	867 (8.1)	453 (8.5)	414 (7.8)	0.027
Chronic liver disease	1235 (11.6)	640 (12.0)	595 (11.2)	0.026
Neuropsychiatric conditions	4586 (43.1)	2550 (47.9)	2036 (38.3)	0.196
Diabetes (type 1 and 2)	382 (3.6)	180 (3.4)	202 (3.8)	0.022
Metabolic disease	1917 (18.0)	926 (17.4)	991 (18.6)	0.032
Cancer	844 (7.9)	435 (8.2)	409 (7.7)	0.018
Hypertension	1766 (16.6)	879 (16.5)	887 (16.7)	0.004
Obesity	899 (8.4)	434 (8.2)	465 (8.7)	0.021
Years on ART, median (IQR)	8.3 (4.3–13.1)	9.0 (5.0–14.2)	7.5 (3.7–12.2)	0.271
Receiving ART				0.077
Yes	9850 (92.6)	4979 (93.6)	4871 (91.6)	
No	790 (7.4)	341 (6.4)	449 (8.4)	

Data are presented as No. (%) unless otherwise indicated.

Abbreviations: ART, antiretroviral therapy; HIV, human immunodeficiency virus; IQR, interquartile range; MSM, men who have sex with men; PWID, people who inject drugs; SMD, standardized mean difference.

^a^Migrants were from Latin America, Western Europe and North America, sub-Saharan Africa, and other places in 3053 (57.4%), 1139 (21.4%), 467 (8.8%), and 661 (12.34%), respectively.

In the matched cohort, fewer migrants received at least 1 SARS-CoV-2 test compared to the non-migrant group (3609 [67.8%] vs 3837 [72.1%], *P* < .0001). However, there was no significant difference in the SARS-CoV-2 diagnosis between the 2 populations (1552 [29.2%] vs 1562 [29.4%], *P* = .847). Regarding SARS-CoV-2 vaccination, migrants had lower complete vaccination (4198 [78.9%] vs 4525 [85.1%], *P* < .0001) and booster doses (2137 [63.0%] vs 2517 [65.5%], *P* = .027) compared to Spanish natives (Table 3). In terms of clinical severity, more migrants were hospitalized for COVID-19 (126 [8.1%] vs 79 [5.1%], *P* < .0001) and admitted to the ICU (45 [2.9%] vs 18 [1.2%], *P* < .0001) compared to their Spanish counterparts. However, we observed comparable length of hospitalization (median, 5.5 [IQR, 2.0–10.0] days vs 4.0 [IQR, 0–10.0] days; *P* = .098) and mortality (3 [0.2%] vs 6 [0.4%]; *P* = .510) between the 2 groups (Table 3).

**Table 3.** Coronavirus Disease 2019 Outcomes and Vaccination Coverage in Propensity Score–Matched Migrants and Non-migrants With Human Immunodeficiency Virus in the PISCIS Cohort, 1 March 2020—30 April 2022

Characteristic	Total (N = 10 640)	Non-migrants (n = 5320)	Migrants (n = 5320)	*P* Value
SARS-CoV-2 testing				<.0001
Yes	7446 (70)	3837 (72.1)	3609 (67.8)	
No	3194 (30)	1483 (27.9)	1711 (32.2)	
SARS-CoV-2 diagnosis				.847
Positive	3114 (29.3)	1562 (29.4)	1552 (29.2)	
Negative	7526 (70.7)	3758 (70.6)	3768 (70.8)	
COVID-19 clinical severity				
Hospital admission (including ICU admissions)	205 (6.6)	79 (5.1)	126 (8.1)	<.0001
Days in hospital, median (IQR)	5.0 (1.0–10.0)	4.0 (0–10.0)	5.5 (2.0–10.0)	.098
ICU admission	63 (2)	18 (1.2)	45 (2.9)	<.0001
COVID-19 deaths	9 (0.3)	6 (0.4)	3 (0.2)	.510
SARS-CoV-2 vaccination				<.0001
Complete vaccination	7230 (68.0)	3840 (72.2)	3390 (63.7)	
Incomplete vaccination	250 (2.3)	134 (2.5)	116 (2.2)	
Unvaccinated	3160 (29.7)	1346 (25.3)	1814 (34.1)	
Booster doses				.027
Yes	4654 (64.4)	2517 (65.5)	2137 (63.0)	
No	2576 (35.6)	1323 (34.5)	1253 (37.0)	

Data are presented as No. (%) unless otherwise indicated.

Abbreviations: COVID-19, coronavirus disease 2019; ICU, intensive care unit; IQR, interquartile range; SARS-CoV-2, severe acute respiratory syndrome coronavirus 2.

**Figure 1.**
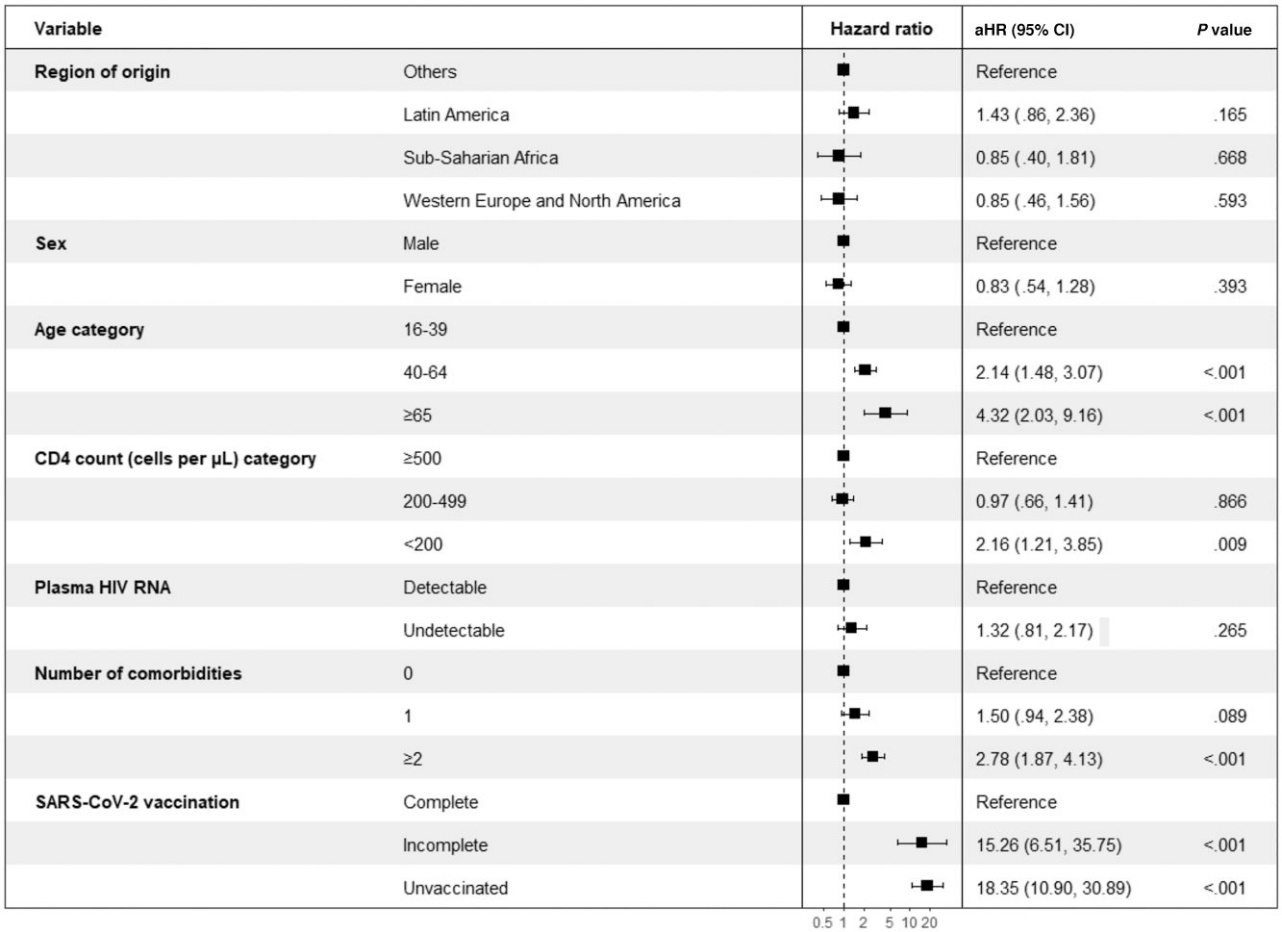
Factors associated with severe coronavirus disease 2019 as calculated using Cox proportional hazards models among migrants with HIV in Catalonia, Spain, 1 March 2020–April 2022. Model adjusted for sex, age, socioeconomic deprivation, plasma HIV RNA viral load (categorized as detectable or undetectable), CD4 cell count (categorized as <200 cells/μL, 200–499 cells/μL, or ≥500 cells/μL), and number of comorbidities. Abbreviations: aHR, adjusted hazard ratio; CI, confidence interval; HIV, human immunodeficiency virus; SARS-CoV-2, severe acute respiratory syndrome coronavirus 2.

**Figure 2.**
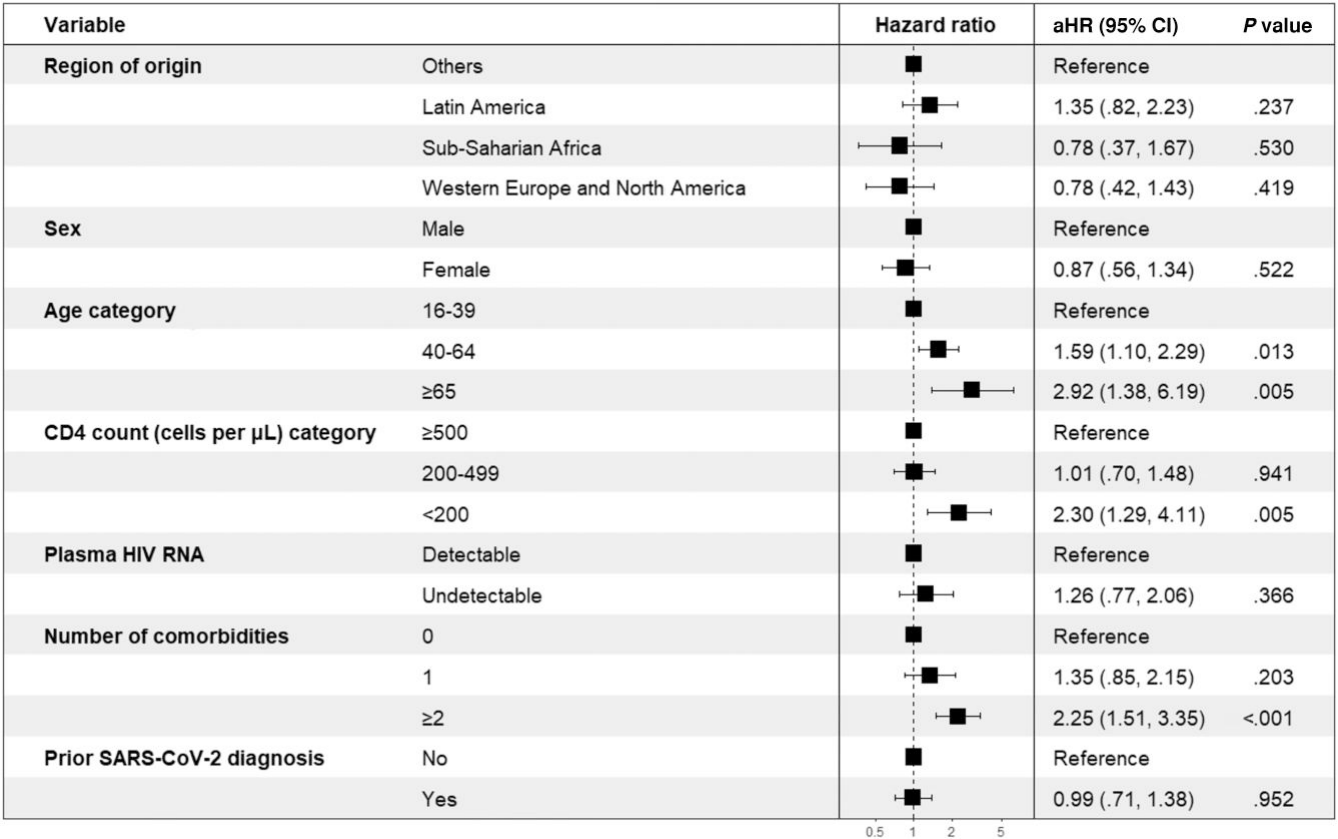
Factors associated with vaccination uptake as calculated using Cox proportional hazards models among migrants with HIV in Catalonia, Spain, 1 March 2020–30 April 2022. Model adjusted for sex, age, socioeconomic deprivation, plasma HIV RNA viral load (categorized as detectable or undetectable), CD4 cell count (categorized as <200 cells/μL, 200–499 cells/μL, or ≥500 cells/μL), number of comorbidities, and prior SARS-CoV-2 diagnosis. Abbreviations: aHR, adjusted hazard ratio; CI, confidence interval; HIV, human immunodeficiency virus; SARS-CoV-2, severe acute respiratory syndrome coronavirus 2.

In multivariable regression analysis involving only the migrant population, there was no significant difference in risk of severe COVID-19 among migrants from Latin America (aHR, 1.35 [95% CI, .82–2.23]), sub-Saharan Africa (aHR, 0.78 [95% CI, .37–1.67]), and Western Europe and North America (aHR, 0.78 [95% CI, .42–1.43]) compared to those from other regions. Factors associated with a higher risk of severe COVID-19 included being aged ≥40 years, having a CD4 count <200 cells/μL, having 2 or more comorbidities, and nonreception or incomplete reception of the SARS-CoV-2 vaccines (Figure 1). In the matched Spanish group, these findings were similar (Supplementary Figure 2). In sensitivity analysis, we excluded SARS-CoV-2 vaccination in the Cox model and the findings were largely consistent except a noteworthy finding: an observed protective effect in migrants from Western Europe and North America (aHR, 0.57 [95% CI, .37–.90]) (Supplementary Figure 3). MWH from Latin America (aHR, 1.39 [95% CI, 1.27–1.51]) and Western Europe and North America (aHR, 1.25 [95% CI, 1.13–1.38]) were more likely to be vaccinated against SARS-CoV-2 than other migrants. In the migrant population, vaccination coverage was also higher among migrants aged ≥40 years, those with undetectable HIV RNA, and those with at least 1 comorbidity (Figure 2).

## DISCUSSION

In this propensity score–matched cohort of PWH in Catalonia, Spain, we found that MWH in Catalonia had higher rates of COVID-19–associated hospitalizations and ICU admissions compared to their Spanish non-migrant counterparts. Additionally, migrants had lower rates of SARS-CoV-2 testing and vaccination coverage, although the rates of SARS-CoV-2 diagnosis were similar between the 2 groups. Migrants who were ≥40 years of age, had a CD4 count of <200 cells/μL, had 2 or more comorbidities, and who were unvaccinated or incompletely vaccinated against SARS-CoV-2 were found to have a significantly increased risk of severe COVID-19.

Migrants with HIV in our study underwent less frequent SARS-CoV-2 testing compared to the host Spanish PWH, indicating potential barriers to accessing testing services. While this is the first study comprehensively evaluating SARS-CoV-2 testing among MWH, a study from the general population in Madrid found similar testing rates between migrants and the host population [7]. Reports from around the world suggest that migrants face health inequalities due to economic, employment, and information-related concerns, limiting their access to healthcare [4, 5]. These factors may partly explain reduced testing rates among migrants. Also, MWH in Catalonia used healthcare services slightly less than non-migrants during the peaks of the COVID-19 pandemic, potentially contributing to the reduced testing [31]. Investigating these access barriers is crucial, as Catalonia offers universal healthcare coverage to all residents regardless of nationality or legal status.

Contrary to findings in the general population of Spain [32] and PWH population in Catalonia [16], our study revealed a similar test positivity rate among migrants. Previous studies have attributed the increased SARS-CoV-2 infection risk in migrants to factors such as crowded living conditions, lower socioeconomic status, sociocultural differences, and essential work that limits the ability to work from home. However, our study did not observe this disparity, suggesting that country of origin or ethnicity alone may not fully explain the differences in SARS-CoV-2 diagnosis between the 2 populations in previous studies [8–12, 16, 32].

We observed that MWH had higher rates of COVID-19–associated hospitalization and ICU admission, even when key confounders such as age, chronic respiratory disease, cardiovascular disease, obesity, diabetes, and malignancies were equal in both groups. These results align with findings in the general population of the UK, where Asian migrants were at an increased risk of hospitalization and ICU admission, accounting for diabetes and cardiovascular disease [33, 34]. Structural discrimination has been identified as a driver of poor clinical outcomes among migrants and minority groups during the COVID-19 pandemic [35]. Although data on this complex challenge among PWH in Spain are limited, other reports have suggested that adherence to control measures for COVID-19 and care-seeking in the presence of COVID-19 symptoms are lower among migrants and ethnic minority groups due to existing health disparities and could contribute to the observed more severe outcomes [35]. Addressing these disparities and implementing policies to reduce health inequalities are crucial in combating the syndemic of HIV and SARS-CoV-2 [36].

We found similar rates of COVID-19–associated mortality between migrants and the non-migrant population, but this finding should be interpreted with caution due to the small number of deaths in both groups. A meta-analysis [2] suggested that certain specific groups of migrants and ethnic minority populations may have a numerically increased risk of COVID-19–related deaths, although not statistically significant (hazard ratio, 1.22 [95% CI, .99–1.50]). Further research is needed to better understand the mortality patterns in these populations.

Contrary to previous reports on specific migrant groups in the general population [2], our adjusted analysis did not identify any particular migrant group at a higher risk of severe COVID-19. Migrants aged 40 years or older were found to have an increased risk of severe COVID-19 in our study. Older age has consistently been identified as a key factor for severe COVID-19. However, a previous study in PWH in Catalonia, Spain [16], found severe outcomes associated with age 75 years or older, suggesting that migrants may experience poor COVID-19 outcomes at a younger age compared to host populations. Additionally, having 2 or more comorbidities was linked to an increased risk of severe COVID-19 among migrants, consistent with studies in PWH [37] and the general population [38].

We observed a correlation between MWH with CD4 count <200 cells/μL and an increased risk of severe COVID-19, consistent with other studies in PWH [16, 24]. However, we did not find an association between unsuppressed HIV viremia and severe COVID-19, contrary to previous reports [16, 24]. Nonreception or incomplete reception of the SARS-CoV-2 vaccine increased the risk of severe COVID-19 by >15-fold, underscoring the importance of promoting vaccination in vulnerable populations. It is crucial for MWH, particularly those with identified risk factors, to adhere to public health guidelines and maintain regular communication with healthcare providers to mitigate their risk of COVID-19 and future pandemics.

Lower vaccination coverage among MWH may stem from access issues, language barriers, mistrust in healthcare systems, or socioeconomic factors [4, 5]. Subpopulations aged ≥40 years, with detectable HIV viremia, and with chronic comorbidities showed higher vaccination rates. These groups are considered more vulnerable to severe COVID-19 among PWH [16]. Promoting vaccinations among migrants to target this subpopulation is crucial, as low vaccination rates not only impact individuals but also pose a public health risk by increasing infection risk for migrants and the wider community [39].

Our study has some notable strengths. To our knowledge, this is the first comprehensive evaluation of COVID-19 among migrants and non-migrants with HIV. The study used adequate propensity score matching to address sociodemographic and clinical differences between the migrant and host populations, enhancing its validity. Also, migrants were underrepresented in previous studies (up to 16% of study population) [2], whereas in our study, migrants comprised 41.8% of the overall cohort.

However, the study has some limitations. First, the findings should be interpreted cautiously because there might be residual confounding arising from migrants generally being younger and having fewer comorbidities, which could impact the comparisons in COVID-19 testing and vaccination. Spain’s initial COVID-19 strategies prioritized testing for symptomatic individuals and those with underlying health conditions. Similarly, the vaccination campaign also factored in occupation, age, and existing health conditions. We also do not report home self-tests for COVID-19, which were available in Spain since July 2021. These differences in testing criteria and vaccination priorities might influence the comparisons drawn between migrant and non-migrant populations. Second, data on smoking, body mass index, and housing, known to affect COVID-19 clinical outcomes, are lacking. Also, the study lacks key variables that might hold higher relevance among migrant populations including nutrition, exposure to pollutants, and mental health status and could significantly influence COVID-19 outcomes. Third, the PISCIS cohort does not capture information on the administrative status of migrants, including whether they are registered, undocumented, or asylum seekers. This hinders tailored responses to the specific needs of different migrant populations. Furthermore, regulations in Spain prohibit the collection of race and ethnicity data, preventing comparison with previous studies. Additionally, information regarding vaccination received outside Catalonia is absent. To avoid underestimating vaccination rates, participants without recent public healthcare service utilization were excluded. In a prior study within this population [31], migrants exhibited a slightly lower utilization of healthcare services compared to non-migrants. This pattern might potentially reduce access to SARS-CoV-2 testing among migrants. While the study mentioned the socioeconomic deprivation index, it did not delve deeper into factors such as income, education level, or employment status, which could potentially influence access to healthcare services and therefore COVID-19 outcomes. Moreover, the socioeconomic deprivation measure is an ecological variable based on an individual's place of residence. A person’s place of residence may indeed not necessarily reflect their socioeconomic deprivation.

In conclusion, this study sheds light on the impact of COVID-19 on MWH compared to their non-migrant counterparts. The study revealed that migrants had lower rates of SARS-CoV-2 testing compared to the non-migrant population. However, the COVID-19 diagnosis rate was similar between the 2 groups. We also found that migrants experienced higher rates of hospitalization and ICU admission due to COVID-19 compared to the host population albeit a similar length of hospitalization and related mortality. Age ≥40 years, CD4 count <200 cells/μL, having ≥2 comorbidities, and nonreceipt/incomplete SARS-CoV-2 vaccination increased severe COVID-19 risk among MWH in Catalonia. Additionally, vaccination coverage was lower among migrants. This study emphasizes the need for comprehensive and inclusive healthcare policies and interventions to address the challenges faced by migrants, especially those with HIV. Improving access to testing, enhancing healthcare delivery, and promoting vaccination equity are crucial steps to mitigate disparities in COVID-19 outcomes and vaccination rates in this vulnerable population. The findings of this study can inform public health policies and interventions to address disparities in future pandemic responses among migrant populations, particularly those with HIV.

## Supplementary Material

ofad693_Supplementary_Data

## Supplementary Data

Supplementary materials are available at *Open Forum Infectious Diseases* online. Consisting of data provided by the authors to benefit the reader, the posted materials are not copyedited and are the sole responsibility of the authors, so questions or comments should be addressed to the corresponding author.

## Notes

***Author contributions.*** D. K. N. and Y. D. conceptualized and designed the study. D. K. N. and Y. D. had full access to all of the study data, verified the data, and take responsibility for the integrity of the data and the accuracy of the data analysis. D. K. N., Y. D., S. M., and J. A. performed the analyses. Acquisition of the financial support for the project was done by J. M. L., and J. R.-U. and D. K. N. wrote the original draft of the paper and incorporated revisions. All authors contributed to the interpretation of results. All authors critically revised and approved the final manuscript.

***Data availability.*** The study protocol is available from Daniel K. Nomah (e-mail: dnomah@igtp.cat). Statistical code for the analysis can be requested from Yesika Diáz, Sergio Moreno-Fornés, and Jordi Aceiton (ydiazr@iconcologia.net, smorenof@iconcologia.net, jaceiton@iconcologia.net). The data for this study are available at the Centre for Epidemiological Studies of Sexually Transmitted Diseases and HIV/AIDS in Catalonia (CEEISCAT), the coordinating center of the PISCIS cohort, and from each of the collaborating hospitals upon request via https://pisciscohort.org/contacte/.

***Patient consent.*** The design of the work has been approved by the Ethics Committee of the Germans Trias i Pujol University Hospital, Badalona, Spain (EO-11-108). Catalan patient data extraction is allowed by of the 203/2015 Decree of the 15th of September, from the Catalan Health Department. Informed consent is therefore not applicable. PISCIS data are owned by each individual patient, and data are eliminated if requested by the hospital or by a patient. Data are pseudo-anonymized before arriving at the coordinating center, and confidentiality is guaranteed in accordance with the provisions of the Regulation (EU) 2016/679 of the European Parliament and of the Council of 27 April 2016 on the protection of natural persons with regarding the processing of personal data and on the free movement of such data and the new national Organic Law of Protection of Personal Data (3/2018), 5th of December, Data Protection and Digital Rights Act. Patient-level information obtained from PADRIS was anonymized and deidentified prior to the analyses.

***Financial support.*** This work was supported by the Fundació La Marató de TV3 (grant number 202117-30-31) and a personal 80:20 research grant from Institut d’Investigacions Biomèdiques August Pi i Sunyer (IDIBAPS), Barcelona, Spain, during 2017–2024 to J. M. M.
