# Supplementary material for: Disparities in Coronavirus Disease 2019 Clinical Outcomes and Vaccination Coverage Among Migrants With Human Immunodeficiency Virus in the PISCIS Cohort: A Population-Based Propensity Score–Matched Analysis
Source: Open Forum Infect Dis. 2024 Jan 5;11(1):ofad693. doi: 10.1093/ofid/ofad693 (PMC10785217; doi:10.1093/ofid/ofad693)
Supplement: ofad693_Supplementary_Data [file ofad693_supplementary_data.zip › Supplimentary_PISCIS_Migrants_COVID.docx]

**Supplementary tables**

**Table S1: Characteristics of migrants and non-migrants LTFU excluded from the study**

|  | **Total PLHIV, n=21480** | **PWH included in study, n=18764** | **Non-migrants LTFU, n=1066** | **Migrants LTFU, n=1650** | **P value*** |
| --- | --- | --- | --- | --- | --- |
| **Characteristic** | n (%) | n (%) | n (%) | n (%) |  |
| **Sex** |  |  |  |  | 0.127 |
| Male | 17679 (82.3) | 15412 (82.1) | 905 (84.9) | 1362 (82.5) |  |
| Female | 3800 (17.7) | 3352 (17.9) | 161 (15.1) | 287 (17.4) |  |
| Missing | 1 (0) | 0 (0) | 0 (0) | 1 (0.1) |  |
| **Age, median (IQR), y** | 44 (36-53) | 45 (37-53) | 42 (35-50) | 38 (32-45) | <0.0001 |
| **Age category, y** |  |  |  |  | <0.0001 |
| 16-39 | 7449 (34.7) | 6125 (32.6) | 421 (39.5) | 903 (54.7) |  |
| 40-64 | 13012 (60.6) | 11653 (62.1) | 623 (58.4) | 736 (44.6) |  |
| ≥65 | 1019 (4.7) | 986 (5.3) | 22 (2.1) | 11 (0.7) |  |
| **Socioeconomic deprivation** |  |  |  |  | 0.054 |
| Least deprived | 10824 (50.4) | 9336 (49.8) | 561 (52.6) | 927 (56.2) |  |
| Mildly deprived | 4034 (18.8) | 3577 (19.1) | 201 (18.9) | 256 (15.5) |  |
| Moderately/severely deprived | 6145 (28.6) | 5427 (28.9) | 279 (26.2) | 439 (26.6) |  |
| Missing | 477 (2.2) | 424 (2.3) | 25 (2.3) | 28 (1.7) |  |
| **HIV acquisition risk group** |  |  |  |  | <0.0001 |
| PWID | 2823 (13.1) | 2422 (12.9) | 272 (25.5) | 129 (7.8) |  |
| MSM | 11491 (53.5) | 10008 (53.3) | 557 (52.3) | 926 (56.1) |  |
| Male heterosexual | 2790 (13.0) | 2482 (13.2) | 82 (7.7) | 226 (13.7) |  |
| Female hetero/homo/bisexual | 2787 (13.0) | 2466 (13.1) | 93 (8.7) | 228 (13.8) |  |
| Other | 600 (2.8) | 538 (2.9) | 16 (1.5) | 46 (2.8) |  |
| Missing | 989 (4.6) | 848 (4.5) | 46 (4.3) | 95 (5.8) |  |
| **Years since HIV diagnosis, median (IQR)** | 10.4 (5.0-17.3) | 10.8 (5.1-17.7) | 10.1 (5.4-17.7) | 7.5 (3.3-11.9) | <0.0001 |
| **CD4 count (cells per µL), category** |  |  |  |  | 0.087 |
| <200 | 960 (4.5) | 743 (4.0) | 92 (8.6) | 125 (7.6) |  |
| 200-499 | 4908 (22.8) | 4027 (21.5) | 322 (30.2) | 559 (33.9) |  |
| ≥500 | 13710 (63.8) | 12169 (64.9) | 627 (58.8) | 914 (55.4) |  |
| Missing | 1902 (8.9) | 1825 (9.7) | 25 (2.3) | 52 (3.2) |  |
| **CD4 count (cells per µL), median (IQR)** | 657 (457-883) | 670 (471-900) | 576 (366-798) | 551 (378-750) | 0.144 |
| **Plasma HIV-RNA** |  |  |  |  | 0.632 |
| Detectable | 3118 (14.5) | 2200 (11.7) | 355 (33.3) | 563 (34.1) |  |
| Undetectable | 16424 (76.5) | 14704 (78.4) | 683 (64.1) | 1037 (62.8) |  |
| Missing | 1938 (9.0) | 1860 (9.9) | 28 (2.6) | 50 (3.0) |  |
| **Number of comorbidities** |  |  |  |  | <0.0001 |
| 0 | 7065 (32.9) | 5430 (28.9) | 579 (54.3) | 1056 (64) |  |
| 1 | 4696 (21.9) | 4160 (22.2) | 217 (20.4) | 319 (19.3) |  |
| ≥2 | 9719 (45.2) | 9174 (48.9) | 270 (25.3) | 275 (16.7) |  |
| **Type of comorbidities** |  |  |  |  |  |
| Respiratory disease | 4004 (18.6) | 3818 (20.3) | 98 (9.2) | 88 (5.3) | <0.0001 |
| Cardiovascular disease | 2929 (13.6) | 2810 (15.0) | 61 (5.7) | 58 (3.5) | 0.008 |
| Autoimmune disease | 2033 (9.5) | 1952 (10.4) | 36 (3.4) | 45 (2.7) | 0.391 |
| Chronic kidney disease | 1699 (7.9) | 1619 (8.6) | 33 (3.1) | 47 (2.8) | 0.798 |
| Chronic liver disease | 3844 (17.9) | 3565 (19.0) | 146 (13.7) | 133 (8.1) | <0.0001 |
| Neuropsychiatric conditions | 9774 (45.5) | 9062 (48.3) | 357 (33.5) | 355 (21.5) | <0.0001 |
| Diabetes (type I and II) | 1028 (4.8) | 985 (5.2) | 24 (2.3) | 19 (1.2) | 0.037 |
| Metabolic disease | 4306 (20.0) | 4124 (22.0) | 91 (8.5) | 91 (5.5) | 0.002 |
| Cancer | 1892 (8.8) | 1778 (9.5) | 56 (5.3) | 58 (3.5) | 0.035 |
| Hypertension | 3816 (17.8) | 3633 (19.4) | 73 (6.8) | 110 (6.7) | 0.915 |
| Obesity | 1819 (8.5) | 1768 (9.4) | 26 (2.4) | 25 (1.5) | 0.112 |

Abbreviations: PWH, people with HIV; LTFU, lost to follow-up; IQR, interquartile range; PWID, people who inject drugs; MSM, men who have sex with men.

*P value comparing migrants and non-migrants lost to follow-up

**Table S2: Baseline characteristics of people with HIV in the PISCIS cohort according to region of origin: March 1, 2020 – April 30, 2022.**

|  | Spanish | Western Europe and Northern America | Latin America | Sub-Saharan Africa | Others | SMD |
| --- | --- | --- | --- | --- | --- | --- |
| Characteristic | 10922 (%) | 1608 (%) | 4557 (%) | 678 (%) | 999 (%) |  |
| **Sex** |  |  |  |  |  | 0.408 |
| Male | 8846 (81) | 1488 (92.5) | 3903 (85.6) | 365 (53.8) | 810 (81.1) |  |
| Female | 2076 (19) | 120 (7.5) | 654 (14.4) | 313 (46.2) | 189 (18.9) |  |
| **Age, median (IQR), y** | 49 (41-56) | 44 (37-52) | 39 (32-46) | 43 (37-50) | 40 (34-47) | 0.405 |
| **Age category, y** |  |  |  |  |  | 0.343 |
| 16-39 | 2464 (22.6) | 532 (33.1) | 2423 (53.2) | 225 (33.2) | 481 (48.1) |  |
| 40-64 | 7643 (70) | 1017 (63.2) | 2063 (45.3) | 431 (63.6) | 499 (49.9) |  |
| ≥65 | 815 (7.5) | 59 (3.7) | 71 (1.6) | 22 (3.2) | 19 (1.9) |  |
| **Socioeconomic deprivation index** | 38.5 (25.6-49.9) | 33.8 (19.3-43.8) | 32.2 (19.3-46.6) | 46.6 (36.2-60.1) | 38.4 (23.7-53.9) | 0.432 |
| **Socioeconomic deprivation category** |  |  |  |  |  | 0.414 |
| Least deprived | 2331 (21.3) | 388 (24.1) | 1044 (22.9) | 160 (23.6) | 237 (23.7) |  |
| Mildly deprived | 6591 (60.3) | 628 (39.1) | 1276 (28) | 301 (44.4) | 378 (37.8) |  |
| Moderately/severely deprived | 3407 (31.2) | 302 (18.8) | 1062 (23.3) | 296 (43.7) | 360 (36) |  |
| Missing | 230 (2.1) | 31 (1.9) | 134 (2.9) | 16 (2.4) | 13 (1.3) |  |
| **HIV acquisition risk group** |  |  |  |  |  | 1.138 |
| PWID | 2087 (19.1) | 138 (8.6) | 67 (1.5) | 12 (1.8) | 118 (11.8) |  |
| MSM | 5174 (47.4) | 1138 (70.8) | 3226 (70.8) | 23 (3.4) | 447 (44.7) |  |
| Male heterosexual | 1483 (13.6) | 124 (7.7) | 422 (9.3) | 272 (40.1) | 181 (18.1) |  |
| Female hetero/homo/bisexual | 1414 (12.9) | 72 (4.5) | 548 (12) | 287 (42.3) | 145 (14.5) |  |
| Other | 289 (2.6) | 12 (0.7) | 155 (3.4) | 56 (8.3) | 26 (2.6) |  |
| Missing | 475 (4.3) | 124 (7.7) | 139 (3.1) | 28 (4.1) | 82 (8.2) |  |
| **Years since HIV diagnosis, median (IQR)** | 13.3 (7.01-20.6) | 9.7 (4.6-16.0) | 7.2 (2.9-12.2) | 10.0 (4.6-14.6) | 6.7 (3.0-11.7) | 0.419 |
| **CD4 count (cells per μL) category** |  |  |  |  |  | 0.240 |
| <200 | 395 (3.6) | 48 (3) | 200 (4.4) | 54 (8) | 46 (4.6) |  |
| 200-499 | 2153 (19.7) | 282 (17.5) | 1137 (25) | 232 (34.2) | 223 (22.3) |  |
| ≥500 | 7293 (66.8) | 1089 (67.7) | 2849 (62.5) | 314 (46.3) | 624 (62.5) |  |
| Missing | 1081 (9.9) | 189 (11.8) | 371 (8.1) | 78 (11.5) | 106 (10.6) |  |
| **CD4 count (cells per μL), median (IQR)** | 698 (490-931) | 704 (510-942.5) | 630.5 (445-840) | 510 (350-720.25) | 659 (446-880) | 0.265 |
| **CD4/CD8 ratio, median (IQR)** | 0.86 (0.57-1.21) | 0.86 (0.6-1.18) | 0.82 (0.54-1.16) | 0.73 (0.43-1.12) | 0.79 (0.52-1.13) | 0.074 |
| Plasma HIV-RNA |  |  |  |  |  | 0.101 |
| Detectable | 1077 (9.9) | 168 (10.4) | 709 (15.6) | 92 (13.6) | 154 (15.4) |  |
| Undetectable | 8709 (79.7) | 1254 (78) | 3505 (76.9) | 500 (73.7) | 736 (73.7) |  |
| Missing | 1136 (10.4) | 186 (11.6) | 343 (7.5) | 86 (12.7) | 109 (10.9) |  |
| Number of comorbidities |  |  |  |  |  | 0.333 |
| 0 | 2000 (18.3) | 592 (36.8) | 2237 (49.1) | 217 (32) | 384 (38.4) |  |
| 1 | 2331 (21.3) | 388 (24.1) | 1044 (22.9) | 160 (23.6) | 237 (23.7) |  |
| ≥2 | 6591 (60.3) | 628 (39.1) | 1276 (28) | 301 (44.4) | 378 (37.8) |  |
| **Type of comorbidities** |  |  |  |  |  |  |
| Respiratory disease | 2812 (25.7) | 259 (16.1) | 512 (11.2) | 79 (11.7) | 156 (15.6) | 0.178 |
| Cardiovascular disease | 2058 (18.8) | 178 (11.1) | 375 (8.2) | 96 (14.2) | 103 (10.3) | 0.150 |
| Autoimmune disease | 1365 (12.5) | 134 (8.3) | 304 (6.7) | 55 (8.1) | 94 (9.4) | 0.089 |
| Chronic kidney disease | 1155 (10.6) | 103 (6.4) | 253 (5.6) | 68 (10) | 40 (4) | 0.136 |
| Chronic liver disease | 2783 (25.5) | 237 (14.7) | 265 (5.8) | 91 (13.4) | 189 (18.9) | 0.256 |
| Neuropsychiatric conditions | 6559 (60.1) | 674 (41.9) | 1241 (27.2) | 188 (27.7) | 400 (40) | 0.337 |
| Diabetes (type I and II) | 735 (6.7) | 49 (3) | 121 (2.7) | 49 (7.2) | 31 (3.1) | 0.120 |
| Metabolic disease | 2955 (27.1) | 261 (16.2) | 635 (13.9) | 129 (19) | 144 (14.4) | 0.157 |
| Cancer | 1288 (11.8) | 145 (9) | 249 (5.5) | 49 (7.2) | 47 (4.7) | 0.132 |
| Hypertension | 2592 (23.7) | 216 (13.4) | 522 (11.5) | 196 (28.9) | 107 (10.7) | 0.253 |
| Obesity | 1220 (11.2) | 80 (5) | 299 (6.6) | 107 (15.8) | 62 (6.2) | 0.181 |
| **Years on ART, median (IQR)** | 10.57 (5.6-16.7) | 6.89 (3.23-11.71) | 5.31 (2.23-10.25) | 7.95 (3.77-12.96) | 5.67 (2.59-9.47) | 0.368 |
| **Receiving ART** |  |  |  |  |  | 0.067 |
| Yes | 9720 (89) | 1360 (84.6) | 3935 (86.4) | 586 (86.4) | 841 (84.2) |  |
| No | 1202 (11) | 248 (15.4) | 622 (13.6) | 92 (13.6) | 158 (15.8) |  |
| **SARS-CoV-2 testing** |  |  |  |  |  | 0.120 |
| Yes | 7802 (71.4) | 1025 (63.7) | 2965 (65.1) | 398 (58.7) | 619 (62) |  |
| No | 3120 (28.6) | 583 (36.3) | 1592 (34.9) | 280 (41.3) | 380 (38) |  |
| **SARS-CoV-2 diagnosis** |  |  |  |  |  | 0.136 |
| Positive | 2877 (26.3) | 384 (23.9) | 1456 (32) | 129 (19) | 228 (22.8) |  |
| Negative | 8045 (73.7) | 1224 (76.1) | 3101 (68) | 549 (81) | 771 (77.2) |  |
| **COVID-19 Clinical severity** |  |  |  |  |  |  |
| Hospital admission (including ICU admissions) | 257 (8.9) | 28 (7.3) | 115 (7.9) | 14 (10.9) | 19 (8.3) | 0.095 |
| Days of hospitalization, median (IQR) | 4.5 (1.0-12.0) | 3.0 (1.0-7.8) | 6.0 (2.0-10.0) | 5.0 (2.5-9.8) | 6.0 (2.0-11.5) | 0.147 |
| ICU admission | 79 (2.7) | 5 (1.3) | 39 (2.7) | 4 (3.1) | 8 (3.5) | 0.063 |
| Death | 42 (1.5) | 0 (0) | 2 (0.1) | 2 (1.6) | 2 (0.9) | 0.107 |
| **SARS-CoV-2 vaccination** |  |  |  |  |  | 0.160 |
| Complete vaccination | 8046 (73.7) | 1032 (64.2) | 2690 (59.0) | 423 (62.4) | 575 (57.6) |  |
| Incomplete vaccination | 249 (2.3) | 38 (2.4) | 125 (2.7) | 17 (2.5) | 28 (2.8) |  |
| Unvaccinated | 2627 (24.1) | 538 (33.5) | 1742 (38.2) | 238 (35.1) | 396 (39.6) |  |
| **Booster doses** |  |  |  |  |  | 0.222 |
| Yes | 5566 (69.2) | 715 (69.3) | 1576 (58.6) | 206 (48.7) | 326 (56.7) |  |
| No | 2480 (30.8) | 317 (30.7) | 1114 (41.4) | 217 (51.3) | 249 (43.3) |  |

Abbreviations: PLHIV, people living with HIV; SARS-CoV-2, severe acute respiratory syndrome coronavirus 2; IQR, interquartile range; PWID, people who inject drugs; MSM, men who have sex with men; ART, antiretroviral therapy; SMD, standardised mean differences; COVID-19, coronavirus disease 2019; ICU, intensive care unit.

**Supplementary figures:**

**
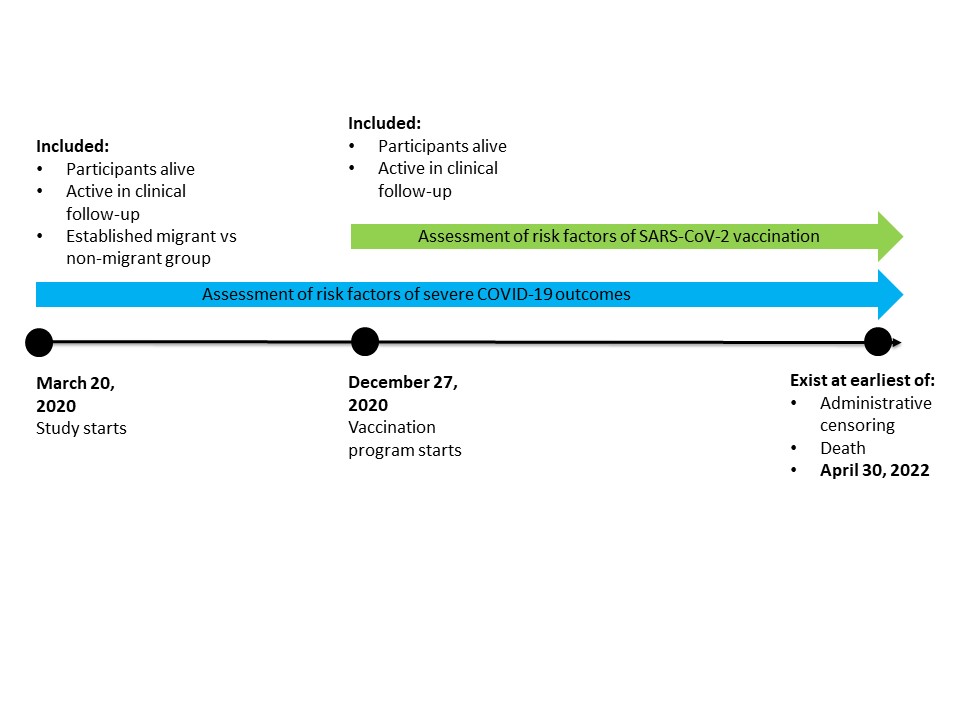
**

**Figure S1: Study design overview.**

**
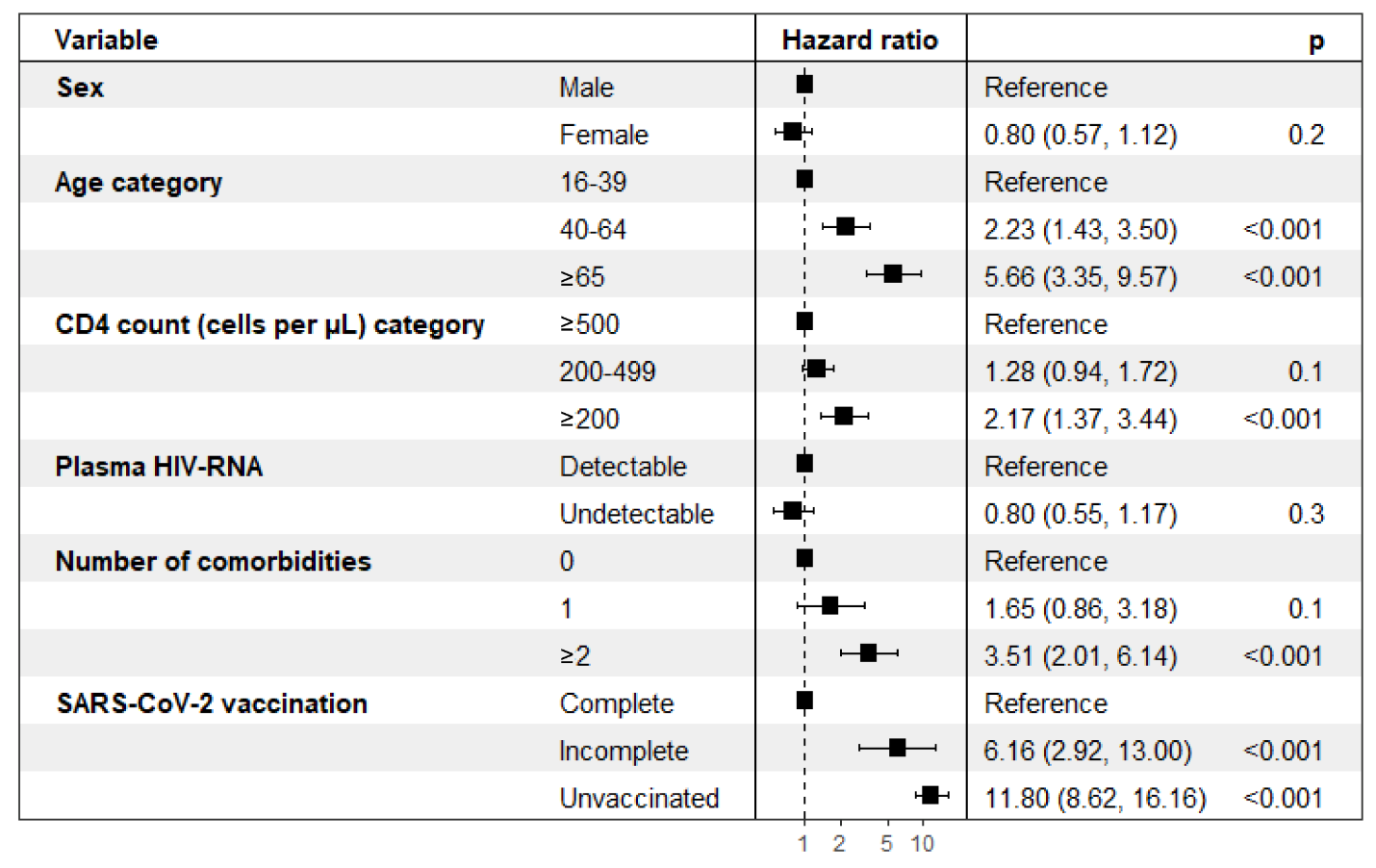
**

**Figure S2: Factors associated with severe COVID-19 as calculated using Cox proportional hazards models among non-migrants (Spanish natives) with HIV in Catalonia, Spain from March 1, 2020 – April 30, 2022.**

Footnotes:

Abbreviations: SARS-CoV-2, severe acute respiratory syndrome coronavirus 2; aHR, adjusted hazard risk.

Model adjusted for sex, age, socioeconomic deprivation, plasma HIV-RNA viral load (categorized detectable and undetectable), CD4 cell count (categorized <200 cells per μL, 200-499 cells per μL, and ≥500 cells per μL), and number of comorbidities.


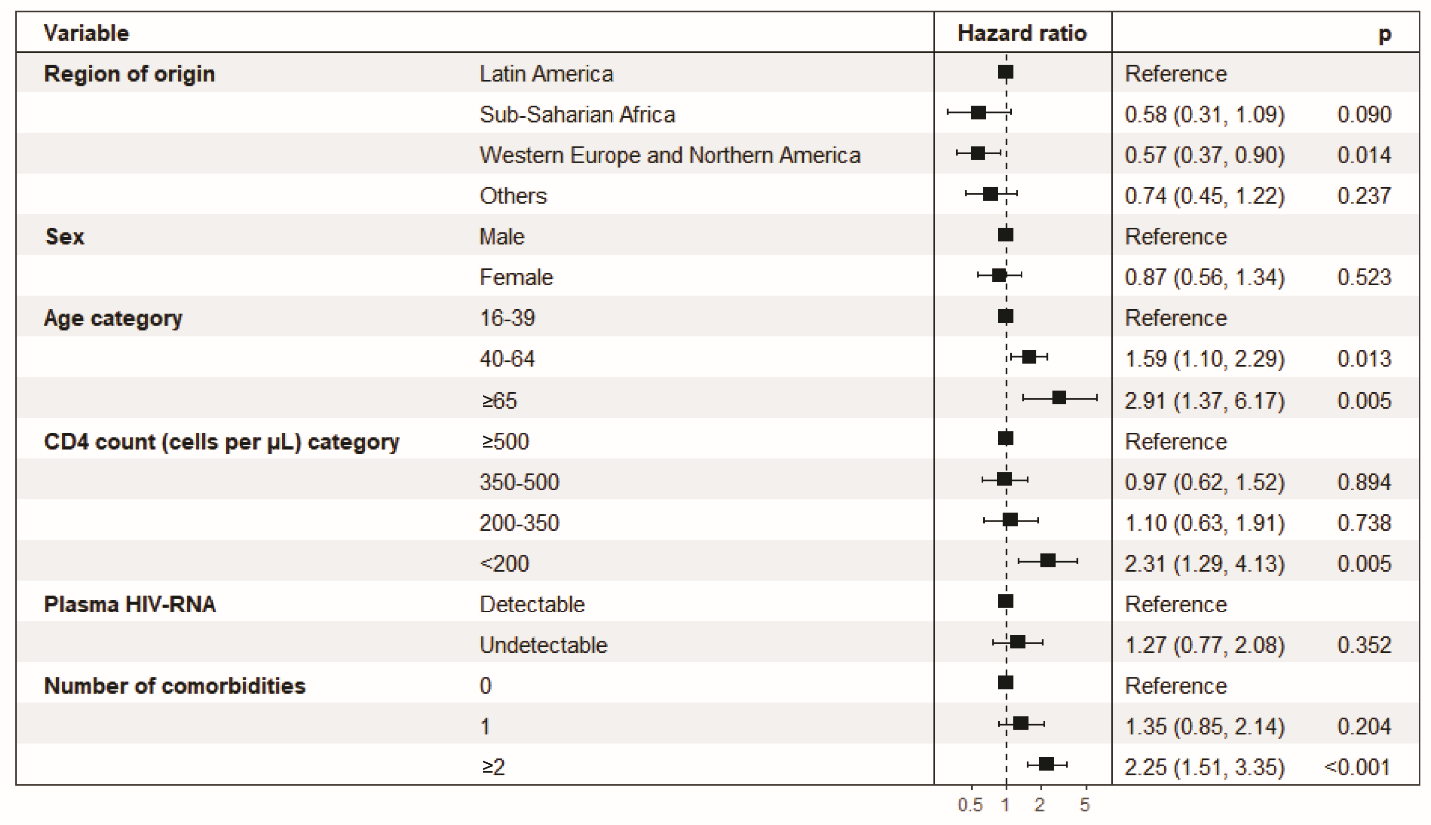


**Figure S3: Factors associated with severe COVID-19 as calculated using Cox proportional hazards models among migrants with HIV in Catalonia, Spain from March 1, 2020 – April 30, 2022. (Model excludes vaccination).**
