# Supplementary material for: Disparities in Coronavirus Disease 2019 Clinical Outcomes and Vaccination Coverage Among Migrants With Human Immunodeficiency Virus in the PISCIS Cohort: A Population-Based Propensity Score–Matched Analysis
Source: Open Forum Infect Dis. 2024 Jan 5;11(1):ofad693. doi: 10.1093/ofid/ofad693 (PMC10785217; doi:10.1093/ofid/ofad693)
Supplement: ofad693_Supplementary_Data [file ofad693_supplementary_data.zip › Appendix_PISCIS_Migrants_COVID.docx]

**Appendix**

**FIGURES**

**Figure A1:** Data linkage process between PISCIS and PADRIS databases


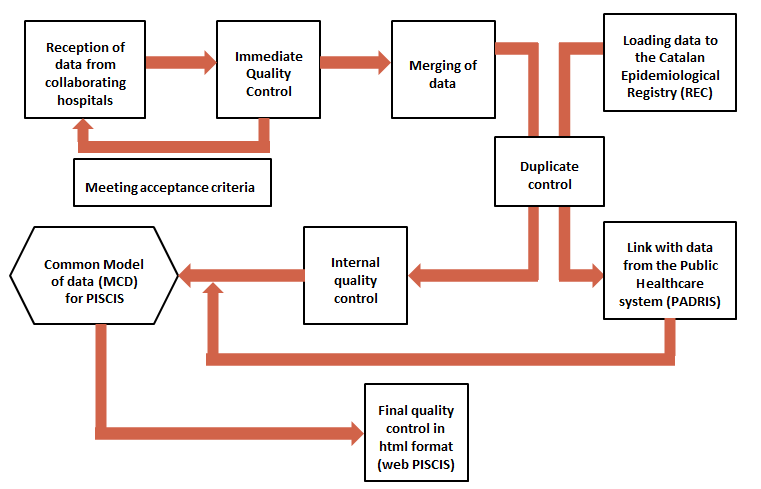


In brief, the above diagram reflects the following process: PISCIS collects data both from patient hospital records and from centralized government health system databases. Every year, it collects computerized hospital record data of HIV patients in follow-up since January 1, 1998. PISCIS data is stored at the coordinating centre, Centre for Epidemiological Studies of Sexually Transmitted Disease and AIDS in Catalonia (CEEISCAT), but each unit’s data is owned by each individual patient, and data is eliminated if requested by the hospital or by a patient. Data is anonymized before arriving at the coordinating centre, and confidentiality is guaranteed in accordance with the provisions of the Regulation (EU) 2016/679 of the European Parliament and Council of 27 April 2016 on the protection of natural persons with regarding the processing of personal data and on the free movement of such data and the new national Organic Law of Protection of Personal Data (3/2018, 5th of December, Data Protection and Digital Rights Act). The PISCIS cohort study has been approved by the ethics committee of the coordinating centre, and Catalan patient data extraction is allowed under the Decree 203/2015, of the 15th of September 2015, from the Catalan Health Department. All participating patients have signed informed consent forms. Catalan general health system data is extracted via the PADRIS program and integrated into PISCIS. PADRIS has several oversight bodies to ensure that the ethical principles of the scientific projects carried out are respected. The structure is made up of the Research Ethics Committee, the Agency for Health Quality and Evaluation of Catalonia (AQuAS) Board of Directors and the Advisory Board, as well as a Supervisory and Operation Committees. Data is stored and managed by AQuAS, but owned by each individual, with ARCO rights (right to access, rectify, cancel or delete, and oppose to commercial use) upheld by the Catalan Data Protection Agency. Data from PADRIS is linked to the PISCIS anonymized ID through the Catalan Epidemiological Registry (REC) within the Catalan Department of Health, as a project that is part of the HIV surveillance system of Catalonia, with complete legal authority. Hospitals upload the patient information and PISCIS ID into the REC, but only the PISCIS ID is seeded with the PADRIS clinical data, so as to maintain confidentiality.

**Figure A2:** Shoenfeld analysis of Hazard proportionality for Cox models of A) severe COVID-19 B) COVID-19 vaccination

A)


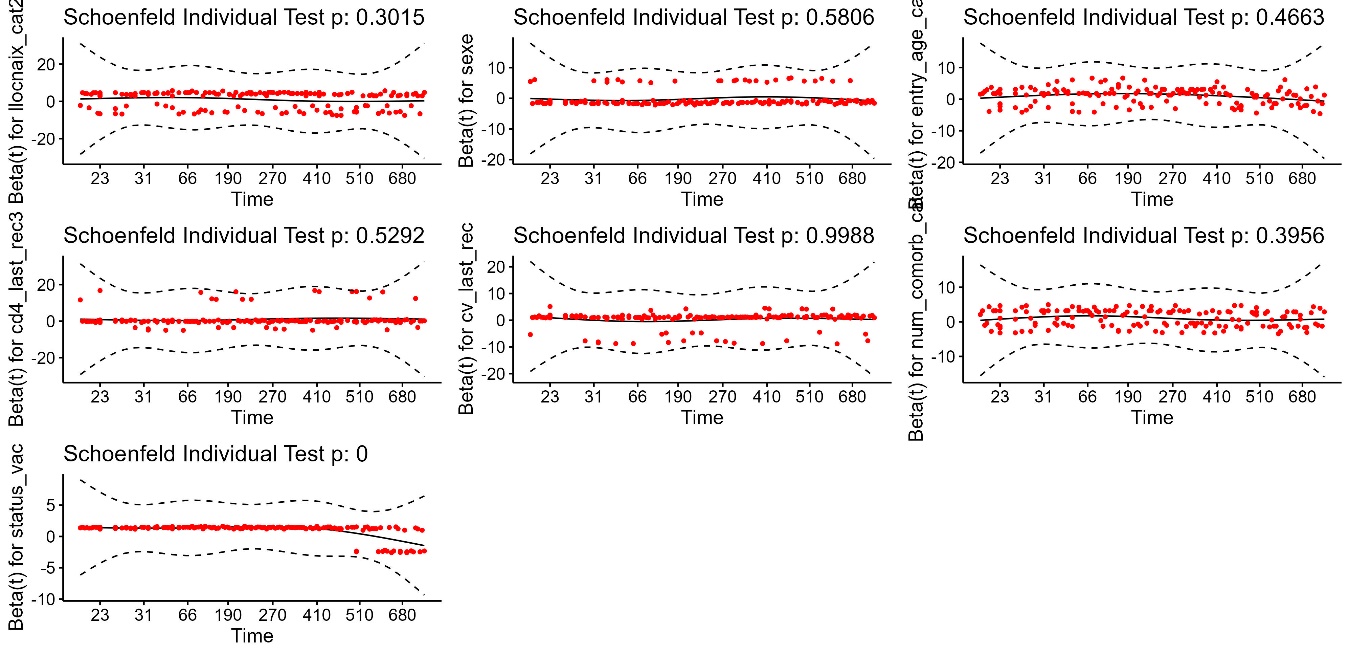


B)


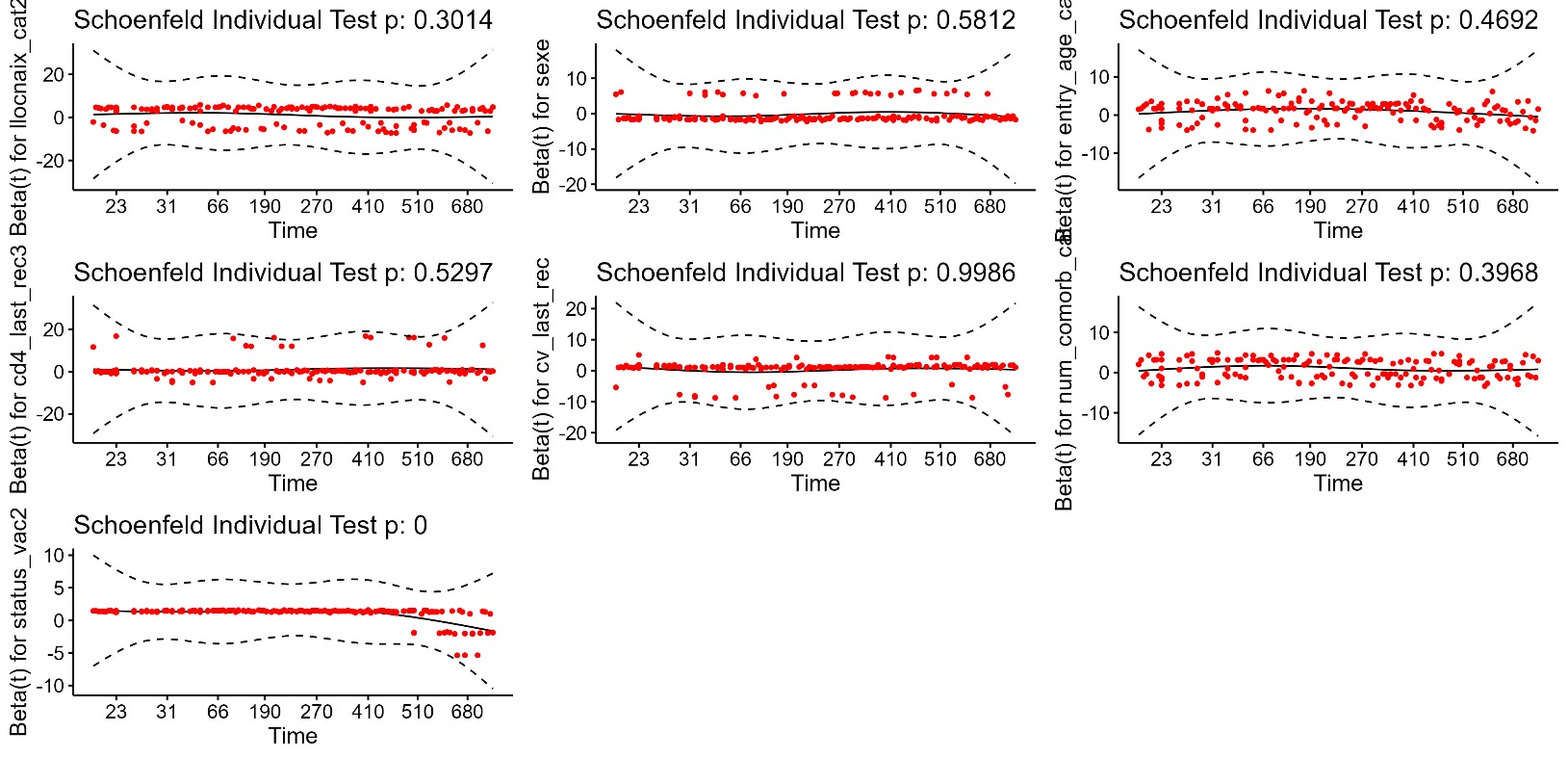


**TABLES**

**Table A1: Eleven groups of the most prevalent chronic comorbidities among HIV-positive individual in Catalonia with corresponding International Classification of Diseases, Ninth and Tenth Revision, Clinical Modification (ICD-9/10-CM)codes.**

| **Disease Group** | **ICD-10-CM** | **Description (ICD-10)** | **ICD-9-CM** | **Description (ICD-9)** |
| --- | --- | --- | --- | --- |
| AUTOIMMUNE | I731 | Thromboangiitis obliterans [Buerger] | 443.1 | Obliterating thromboangeitis (Buerger's disease) |
| AUTOIMMUNE | L10 | Pemphigus |  |  |
| AUTOIMMUNE | L12 | Pemphigoid |  |  |
| AUTOIMMUNE | L40 | Psoriasis |  |  |
| AUTOIMMUNE | L41 | Parapsoriasis |  |  |
| AUTOIMMUNE | L93 | Lupus erythematosus |  |  |
| AUTOIMMUNE | L94 | Other localized connective tissue disorders |  |  |
| AUTOIMMUNE | L95 | Vasculitis limited to skin, not elsewhere classified |  |  |
| AUTOIMMUNE | M30 | Polyarteritis nodosa and related conditions |  |  |
| AUTOIMMUNE | M31 | Other necrotizing vasculopathies |  |  |
| AUTOIMMUNE | M32 | Systemic lupus erythematosus |  |  |
| AUTOIMMUNE | M33 | Dermatopolymyositis |  |  |
| AUTOIMMUNE | M34 | Systemic sclerosis |  |  |
| AUTOIMMUNE | M35 | Other systemic involvement of connective tissue |  |  |
| AUTOIMMUNE | M36 | Systemic disorders of connective tissue in diseases classified elsewhere |  |  |
| AUTOIMMUNE | M023 | Reiter disease |  |  |
| AUTOIMMUNE | M05 | Seropositive rheumatoid arthritis |  |  |
| AUTOIMMUNE | M06 | Other rheumatoid arthritis |  |  |
| AUTOIMMUNE | M07 | Psoriatic and enteropathic arthropathies |  |  |
| AUTOIMMUNE | M08 | Juvenile arthritis |  |  |
| AUTOIMMUNE | M09 | Juvenile arthritis in diseases classified elsewhere |  |  |
| AUTOIMMUNE | M10 | Gout |  |  |
| AUTOIMMUNE | M11 | Other crystal arthropathies |  |  |
| AUTOIMMUNE | M12 | Other specific arthropathies |  |  |
| AUTOIMMUNE | M13 | Other arthritis |  |  |
| AUTOIMMUNE | M14 | Arthropathies in other diseases classified elsewhere |  |  |
| AUTOIMMUNE | M45 | Ankylosing spondylitis |  |  |
| AUTOIMMUNE | M460 | Spinal enthesopathy |  |  |
| AUTOIMMUNE | M461 | Sacroiliitis, not elsewhere classified | 720.2 | Sacroiliitis, not elsewhere classified |
| AUTOIMMUNE | M468 | Other specified inflammatory spondylopathies |  |  |
| AUTOIMMUNE | M469 | Inflammatory spondylopathy, unspecified |  |  |
| AUTOIMMUNE | K50 | Crohn disease [regional enteritis] |  |  |
| AUTOIMMUNE | K51 | Ulcerative colitis |  |  |
| AUTOIMMUNE | G35 | Multiple sclerosis | 340 | Multiple sclerosis |
| CANCER | C81 | Hodgkin lymphoma |  |  |
| CANCER | C82 | Follicular lymphoma |  |  |
| CANCER | C83 | Non-follicular lymphoma |  |  |
| CANCER | C84 | Mature T/NK-cell lymphomas |  |  |
| CANCER | C85 | Other and unspecified types of non-Hodgkin lymphoma |  |  |
| CANCER | C86 | Other specified types of T/NK-cell lymphoma |  |  |
| CANCER | C88 | Malignant immunoproliferative diseases |  |  |
| CANCER | C90 | Multiple myeloma and malignant plasma cell neoplasms |  |  |
| CANCER | C91 | Lymphoid leukaemia |  |  |
| CANCER | C92 | Myeloid leukaemia |  |  |
| CANCER | C93 | Monocytic leukaemia |  |  |
| CANCER | C94 | Other leukaemias of specified cell type |  |  |
| CANCER | C95 | Leukaemia of unspecified cell type |  |  |
| CANCER | C96 | Other and unspecified malignant neoplasms of lymphoid, haematopoietic and related tissue |  |  |
| CANCER | C | Malignant neoplasms |  |  |
| CANCER | D00 | Carcinoma in situ of oral cavity, oesophagus and stomach |  |  |
| CANCER | D01 | Carcinoma in situ of other and unspecified digestive organs |  |  |
| CANCER | D02 | Carcinoma in situ of middle ear and respiratory system |  |  |
| CANCER | D03 | Melanoma in situ |  |  |
| CANCER | D04 | Carcinoma in situ of skin |  |  |
| CANCER | D05 | Carcinoma in situ of breast |  |  |
| CANCER | D06 | Carcinoma in situ of cervix uteri |  |  |
| CANCER | D07 | Carcinoma in situ of other and unspecified genital organs |  |  |
| CANCER | D09 | Carcinoma in situ of other and unspecified sites |  |  |
| CANCER | D320 | Benign neoplasm: Cerebral meninges | 225.2 | Benign neoplasm of cerebral meninges |
| CANCER | D321 | Benign neoplasm: Spinal meninges | 225.4 | Benign neoplasm of spinal meninges |
| CANCER | D329 | Benign neoplasm: Meninges, unspecified |  |  |
| CANCER | D330 | Benign neoplasm: Brain, supratentorial | 225.0 | Benign neoplasm of brain and other parts of nervous system |
| CANCER | D331 | Benign neoplasm: Brain, infratentorial |  |  |
| CANCER | D332 | Benign neoplasm: Brain, unspecified |  |  |
| CANCER | D333 | Benign neoplasm: Cranial nerves | 225.1 | Benign neoplasm of cranial nerves convert |
| CANCER | D334 | Benign neoplasm: Spinal cord | 225.3 | Benign neoplasm of spinal cord |
| CANCER | Q85 | Phakomatoses, not elsewhere classified |  |  |
| CANCER | C81 | Hodgkin lymphoma |  |  |
| CANCER | C82 | Follicular lymphoma |  |  |
| CANCER | C83 | Non-follicular lymphoma |  |  |
| CANCER | C84 | Mature T/NK-cell lymphomas |  |  |
| CANCER | C85 | Other and unspecified types of non-Hodgkin lymphoma |  |  |
| CANCER | C86 | Other specified types of T/NK-cell lymphoma |  |  |
| CANCER | C88 | Malignant immunoproliferative diseases |  |  |
| CANCER | C90 | Multiple myeloma and malignant plasma cell neoplasms |  |  |
| CANCER | C91 | Lymphoid leukaemia |  |  |
| CANCER | C92 | Myeloid leukaemia |  |  |
| CANCER | C93 | Monocytic leukaemia |  |  |
| CANCER | C94 | Other leukaemias of specified cell type |  |  |
| CANCER | C95 | Leukaemia of unspecified cell type |  |  |
| CANCER | C96 | Other and unspecified malignant neoplasms of lymphoid, haematopoietic and related tissue |  |  |
| CARDIOVASCULAR | I48 | Atrial fibrillation and flutter |  |  |
| CARDIOVASCULAR | I441 | Atrioventricular block, second degree | 426.12 | Mobitz (type) II atrioventricular block |
| CARDIOVASCULAR | I441 | Atrioventricular block, second degree | 426.13 | Other second degree atrioventricular block |
| CARDIOVASCULAR | I442 | Atrioventricular block, complete | 426.0 | Atrioventricular block, complete |
| CARDIOVASCULAR | I443 | Other and unspecified atrioventricular block |  |  |
| CARDIOVASCULAR | I453 | Trifascicular block | 426.54 | Trifascicular block |
| CARDIOVASCULAR | I455 | Other specified heart block | 426.6 | Other heart block |
| CARDIOVASCULAR | Z950 | Presence of cardiac pacemaker | V45.01 | Status cardiac pacemaker |
| CARDIOVASCULAR | I05 | Rheumatic mitral valve diseases |  |  |
| CARDIOVASCULAR | I06 | Rheumatic aortic valve diseases |  |  |
| CARDIOVASCULAR | I07 | Rheumatic tricuspid valve diseases |  |  |
| CARDIOVASCULAR | I08 | Multiple valve diseases |  |  |
| CARDIOVASCULAR | I091 | Rheumatic diseases of endocardium, valve unspecified | 397.9 | Rheumatic diseases of endocardium, valve unspecified. |
| CARDIOVASCULAR | I098 | Other specified rheumatic heart diseases |  |  |
| CARDIOVASCULAR | I34 | Nonrheumatic mitral valve disorders |  |  |
| CARDIOVASCULAR | I35 | Nonrheumatic aortic valve disorders |  |  |
| CARDIOVASCULAR | I36 | Nonrheumatic tricuspid valve disorders |  |  |
| CARDIOVASCULAR | I37 | Pulmonary valve disorders |  |  |
| CARDIOVASCULAR | I38 | Endocarditis, valve unspecified | 424.90 | Endocarditis, valve unspecified, unspecified cause. |
| CARDIOVASCULAR | I38 | Endocarditis, valve unspecified | 424.99 | Endocarditis, valve unspecified. |
| CARDIOVASCULAR | I390 | Mitral valve disorders in diseases classified elsewhere |  |  |
| CARDIOVASCULAR | I391 | Aortic valve disorders in diseases classified elsewhere |  |  |
| CARDIOVASCULAR | I392 | Tricuspid valve disorders in diseases classified elsewhere |  |  |
| CARDIOVASCULAR | I393 | Pulmonary valve disorders in diseases classified elsewhere |  |  |
| CARDIOVASCULAR | I394 | Multiple valve disorders in diseases classified elsewhere |  |  |
| CARDIOVASCULAR | Q22 | Congenital malformations of pulmonary and tricuspid valves |  |  |
| CARDIOVASCULAR | Q23 | Congenital malformations of aortic and mitral valves |  |  |
| CARDIOVASCULAR | Z952 | Presence of prosthetic heart valve | V43.3 | Heart valve replaced by other means |
| CARDIOVASCULAR | Z953 | Presence of xenogenic heart valve | V42.2 | Heart valve replaced by transplant |
| CARDIOVASCULAR | Z954 | Presence of other heart-valve replacement | V42.2 |  |
| CARDIOVASCULAR | G45 | Transient cerebral ischaemic attacks and related syndromes |  |  |
| CARDIOVASCULAR | G46 | Vascular syndromes of brain in cerebrovascular diseases |  |  |
| CARDIOVASCULAR | I60 | Subarachnoid haemorrhage |  |  |
| CARDIOVASCULAR | I61 | Intracerebral haemorrhage |  |  |
| CARDIOVASCULAR | I62 | Other nontraumatic intracranial haemorrhage |  |  |
| CARDIOVASCULAR | I63 | Cerebral infarction |  |  |
| CARDIOVASCULAR | I64 | Stroke, not specified as haemorrhage or infarction |  |  |
| CARDIOVASCULAR | I67 | Other cerebrovascular diseases |  |  |
| CARDIOVASCULAR | I69 | Sequelae of cerebrovascular disease |  |  |
| CARDIOVASCULAR | I110 | Hypertensive heart disease with (congestive) heart failure | 402.01 | Malignant hypertensive heart disease with heart failure |
| CARDIOVASCULAR | I110 | Hypertensive heart disease with (congestive) heart failure | 402.11 | Malignant hypertensive heart disease with heart failure |
| CARDIOVASCULAR | I110 | Hypertensive heart disease with (congestive) heart failure | 402.91 | Unspecified hypertensive heart disease |
| CARDIOVASCULAR | I130 | Hypertensive heart and renal disease with (congestive) heart failure | 404.01 | Hypertensive heart and renal disease, with congestive heart failure, malignant |
| CARDIOVASCULAR | I130 | Hypertensive heart and renal disease with (congestive) heart failure | 404.11 | Hypertensive heart and chronic kidney disease with heart failure and stage 1 through stage 4 chronic kidney disease. |
| CARDIOVASCULAR | I130 | Hypertensive heart and renal disease with (congestive) heart failure | 404.91 | Hypertensive heart and chronic kidney disease with heart failure and stage 1 through stage 4 chronic kidney disease |
| CARDIOVASCULAR | I132 | Hypertensive heart and renal disease with both (congestive) heart failure and renal failure | 404.03 | Hypertensive HF and CKD- Kidney Failure |
| CARDIOVASCULAR | I132 | Hypertensive heart and renal disease with both (congestive) heart failure and renal failure | 404.13 | Hypertensive heart and chronic kidney disease with heart failure and with stage 5 chronic kidney disease |
| CARDIOVASCULAR | I132 | Hypertensive heart and renal disease with both (congestive) heart failure and renal failure | 404.93 | Unspecified w/chf and renal failure |
| CARDIOVASCULAR | I27 | Other pulmonary heart diseases |  |  |
| CARDIOVASCULAR | I280 | Arteriovenous fistula of pulmonary vessels | 417.0 | Arteriovenous fistula of pulmonary vessels |
| CARDIOVASCULAR | I42 | Cardiomyopathy |  |  |
| CARDIOVASCULAR | I43 | Cardiomyopathy in diseases classified elsewhere | 425.8 | Cardiomyopathy in other diseases classified elsewhere |
| CARDIOVASCULAR | I50 | Heart failure |  |  |
| CARDIOVASCULAR | I515 | Myocardial degeneration | 429.1 | Myocardial degeneration |
| CARDIOVASCULAR | I517 | Cardiomegaly | 429.3 | Cardiomegaly |
| CARDIOVASCULAR | I528 | Other heart disorders in other diseases classified elsewhere |  |  |
| CARDIOVASCULAR | Z941 | Heart transplant status | V42.1 | Heart transplant status |
| CARDIOVASCULAR | Z943 | Heart and lungs transplant status | V42.1 |  |
| CARDIOVASCULAR | Z943 | Heart and lungs transplant status | V42.6 | Lung transplant status |
| CARDIOVASCULAR | I20 | Angina pectoris |  |  |
| CARDIOVASCULAR | I21 | Acute myocardial infarction |  |  |
| CARDIOVASCULAR | I22 | Subsequent myocardial infarction |  |  |
| CARDIOVASCULAR | I24 | Other acute ischaemic heart diseases |  |  |
| CARDIOVASCULAR | I25 | Chronic ischaemic heart disease |  |  |
| CARDIOVASCULAR | Z951 | Presence of aortocoronary bypass graft | V45.81 | Aortocoronary bypass status |
| CARDIOVASCULAR | Z955 | Presence of coronary angioplasty implant and graft | V45.82 | Ercutaneous transluminal coronary angioplasty status |
| CARDIOVASCULAR | I09 | Other rheumatic heart diseases |  |  |
| CARDIOVASCULAR | I281 | Aneurysm of pulmonary artery | 417.1 | Aneurysm of pulmonary artery |
| CARDIOVASCULAR | I310 | Chronic adhesive pericarditis | 423.1 | Adhesive pericarditis |
| CARDIOVASCULAR | I311 | Chronic constrictive pericarditis | 423.2 | Constrictive pericarditis. |
| CARDIOVASCULAR | I456 | Pre-excitation syndrome | 426.7 | Anomalous atrioventricular excitation. |
| CARDIOVASCULAR | I456 | Pre-excitation syndrome | 426.81 | Lown-Ganong-Levine syndrome. |
| CARDIOVASCULAR | I495 | Sick sinus syndrome | 427.81 | Sinoatrial node dysfunction |
| CARDIOVASCULAR | I498 | Other specified cardiac arrhythmias | 427.89 | Other specified cardiac dysrhythmias |
| CARDIOVASCULAR | I70 | Atherosclerosis |  |  |
| CARDIOVASCULAR | I71 | Aortic aneurysm and dissection |  |  |
| CARDIOVASCULAR | I72 | Other aneurysm and dissection |  |  |
| CARDIOVASCULAR | I790 | Aneurysm of aorta in diseases classified elsewhere | 441.9 | Aortic aneurysm of unspecified site without mention of rupture |
| CARDIOVASCULAR | I791 | Aortitis in diseases classified elsewhere | 443.81 | Peripheral angiopathy in diseases classified elsewhere |
| CARDIOVASCULAR | I950 | Idiopathic hypotension | 458.1 | Chronic hypotension |
| CARDIOVASCULAR | I951 | Orthostatic hypotension | 458.0 | Orthostatic hypotension |
| CARDIOVASCULAR | I958 | Other hypotension |  |  |
| CARDIOVASCULAR | Q20 | Congenital malformations of cardiac chambers and connections |  |  |
| CARDIOVASCULAR | Q21 | Congenital malformations of cardiac septa |  |  |
| CARDIOVASCULAR | Q24 | Other congenital malformations of heart |  |  |
| CARDIOVASCULAR | Q25 | Congenital malformations of great arteries |  |  |
| CARDIOVASCULAR | Q26 | Congenital malformations of great veins |  |  |
| CARDIOVASCULAR | Q27 | Other congenital malformations of peripheral vascular system |  |  |
| CARDIOVASCULAR | Q28 | Other congenital malformations of circulatory system |  |  |
| CARDIOVASCULAR | Z958 | Presence of other cardiac and vascular implants and grafts |  |  |
| CARDIOVASCULAR | Z959 | Presence of cardiac and vascular implant and graft, unspecified | V45.00 | Cardiac device in situ |
| CARDIOVASCULAR | I091 | Rheumatic diseases of endocardium, valve unspecified | 397.9 | Rheumatic diseases of endocardium, valve unspecified |
| CARDIOVASCULAR | I098 | Other specified rheumatic heart diseases |  |  |
| CARDIOVASCULAR | I702 | Atherosclerosis of arteries of extremities |  |  |
| CARDIOVASCULAR | I780 | Hereditary haemorrhagic telangiectasia | 448.0 | Hereditary hemorrhagic telangiectasia. |
| CARDIOVASCULAR | I83 | Varicose veins of lower extremities |  |  |
| CARDIOVASCULAR | I87 | Other disorders of veins |  |  |
| CARDIOVASCULAR | I89 | Other noninfective disorders of lymphatic vessels and lymph nodes |  |  |
| CARDIOVASCULAR | I972 | Postmastectomy lymphoedema syndrome | 457.0 | Postmastectomy lymphedema syndrome |
| CARDIOVASCULAR | Q820 | Hereditary lymphoedema | 757.0 | Hereditary edema of legs |
| HYPERTENSION | I10 | Essential (primary) hypertension | 401.0 | Malignant essential hypertension |
| HYPERTENSION | I10 | Essential (primary) hypertension | 401.1 | Benign essential hypertension |
| HYPERTENSION | I10 | Essential (primary) hypertension | 401.9 | Unspecified essential hypertension |
| HYPERTENSION | I11 | Hypertensive heart disease |  |  |
| HYPERTENSION | I12 | Hypertensive renal disease |  |  |
| HYPERTENSION | I13 | Hypertensive heart and renal disease |  |  |
| HYPERTENSION | I15 | Secondary hypertension |  |  |
| CHRONIC KIDNEY DISEASES | I120 | Hypertensive renal disease with renal failure | 403.01 | Hypertensive chronic kidney disease, malignant, with chronic kidney disease stage V or end stage renal disease |
| CHRONIC KIDNEY DISEASES | I120 | Hypertensive renal disease with renal failure | 403.11 | Hypertensive chronic kidney disease, benign, with chronic kidney disease stage v or end stage renal disease |
| CHRONIC KIDNEY DISEASES | I120 | Hypertensive renal disease with renal failure | 403.91 | Hypertensive chronic kidney disease, unspecified, with chronic kidney disease stage V or end stage renal disease |
| CHRONIC KIDNEY DISEASES | I130 | Hypertensive heart and renal disease with (congestive) heart failure | 404.01 | Hypertensive heart and renal disease, with congestive heart failure, malignant |
| CHRONIC KIDNEY DISEASES | I130 | Hypertensive heart and renal disease with (congestive) heart failure | 404.11 | Hypertensive heart and chronic kidney disease with heart failure and stage 1 through stage 4 chronic kidney disease, or unspecified chronic kidney disease |
| CHRONIC KIDNEY DISEASES | I130 | Hypertensive heart and renal disease with (congestive) heart failure | 404.91 | Hypertensive heart and chronic kidney disease with heart failure and stage 1 through stage 4 chronic kidney disease, or unspecified chronic kidney disease |
| CHRONIC KIDNEY DISEASES | I131 | Hypertensive heart and renal disease with renal failure |  |  |
| CHRONIC KIDNEY DISEASES | I132 | Hypertensive heart and renal disease with both (congestive) heart failure and renal failure | 404.03 | Hypertensive heart and chronic kidney disease with heart failure and stage 1 through stage 4 chronic kidney disease, or unspecified chronic kidney disease |
| CHRONIC KIDNEY DISEASES | I132 | Hypertensive heart and renal disease with both (congestive) heart failure and renal failure | 404.13 | Hypertensive HF and CKD – Kidney Failure |
| CHRONIC KIDNEY DISEASES | I132 | Hypertensive heart and renal disease with both (congestive) heart failure and renal failure | 404.93 | Hypertensive HF and CKD – Kidney Failure |
| CHRONIC KIDNEY DISEASES | I139 | Hypertensive heart and renal disease, unspecified |  |  |
| CHRONIC KIDNEY DISEASES | N01 | Rapidly progressive nephritic syndrome |  |  |
| CHRONIC KIDNEY DISEASES | N03 | Chronic nephritic syndrome |  |  |
| CHRONIC KIDNEY DISEASES | N04 | Nephrotic syndrome |  |  |
| CHRONIC KIDNEY DISEASES | N05 | Unspecified nephritic syndrome |  |  |
| CHRONIC KIDNEY DISEASES | N07 | Hereditary nephropathy, not elsewhere classified |  |  |
| CHRONIC KIDNEY DISEASES | N08 | Glomerular disorders in diseases classified elsewhere | 583.81 | Nephritis and nephropathy, not specified as acute or chronic, in diseases classified elsewhere |
| CHRONIC KIDNEY DISEASES | N11 | Chronic tubulo-interstitial nephritis |  |  |
| CHRONIC KIDNEY DISEASES | N183 | Chronic kidney disease, stage 3 | 585.3 | Chronic kidney disease, stage 3 (moderate) |
| CHRONIC KIDNEY DISEASES | N184 | Chronic kidney disease, stage 4 | 585.4 | Chronic kidney disease, Stage IV (severe) |
| CHRONIC KIDNEY DISEASES | N185 | Chronic kidney disease, stage 5 | 585.5 | Chronic kidney disease, stage v |
| CHRONIC KIDNEY DISEASES | N189 | Chronic kidney disease, unspecified | 585.9 | Chronic kidney disease, unspecified |
| CHRONIC KIDNEY DISEASES | Q60 | Renal agenesis and other reduction defects of kidney |  |  |
| CHRONIC KIDNEY DISEASES | Q611 | Polycystic kidney, autosomal recessive |  |  |
| CHRONIC KIDNEY DISEASES | Q612 | Polycystic kidney, autosomal dominant | 753.13 | Polycystic kidney, autosomal dominant |
| CHRONIC KIDNEY DISEASES | Q613 | Polycystic kidney, unspecified | 753.12 | Polycystic kidney, unspecified type |
| CHRONIC KIDNEY DISEASES | Q614 | Renal dysplasia | 753.15 | Renal dysplasia |
| CHRONIC KIDNEY DISEASES | Q615 | Medullary cystic kidney | 753.16 | Medullary cystic kidney |
| CHRONIC KIDNEY DISEASES | Q615 | Medullary cystic kidney | 753.17 | Medullary sponge kidney |
| CHRONIC KIDNEY DISEASES | Q618 | Other cystic kidney diseases | 753.19 | Other specified cystic kidney disease |
| CHRONIC KIDNEY DISEASES | Q619 | Cystic kidney disease, unspecified | 753.10 | Cystic kidney disease, unspecified |
| CHRONIC KIDNEY DISEASES | Z905 | Acquired absence of kidney | V45.73 | Acquired absence kidney |
| CHRONIC KIDNEY DISEASES | Z940 | Kidney transplant status | V42.0 | Organ or tissue replaced by transplant |
| CHRONIC LIVER DISEASES | B18 | Chronic viral hepatitis |  |  |
| CHRONIC LIVER DISEASES | K70 | Alcoholic liver disease |  |  |
| CHRONIC LIVER DISEASES | K713 | Toxic liver disease with chronic persistent hepatitis | 573.3 | Hepatitis, unspecified |
| CHRONIC LIVER DISEASES | K714 | Toxic liver disease with chronic lobular hepatitis | 573.3 | Hepatitis, unspecified |
| CHRONIC LIVER DISEASES | K715 | Toxic liver disease with chronic active hepatitis |  |  |
| CHRONIC LIVER DISEASES | K717 | Toxic liver disease with fibrosis and cirrhosis of liver | 573.3 | Hepatitis, unspecified |
| CHRONIC LIVER DISEASES | K721 | Chronic hepatic failure |  |  |
| CHRONIC LIVER DISEASES | K73 | Chronic hepatitis, not elsewhere classified |  |  |
| CHRONIC LIVER DISEASES | K74 | Fibrosis and cirrhosis of liver |  |  |
| CHRONIC LIVER DISEASES | K753 | Granulomatous hepatitis, not elsewhere classified | 573.3 | Hepatitis, unspecified |
| CHRONIC LIVER DISEASES | K754 | Autoimmune hepatitis | 571.42 | Chronic persistent hepatitis |
| CHRONIC LIVER DISEASES | K758 | Other specified inflammatory liver diseases |  |  |
| CHRONIC LIVER DISEASES | K761 | Chronic passive congestion of liver | 573.0 | Chronic passive congestion of liver |
| CHRONIC LIVER DISEASES | K761 | Chronic passive congestion of liver | 573.8 | Other disorders of liver |
| CHRONIC LIVER DISEASES | K766 | Portal hypertension | 572.3 | Portal hypertension |
| CHRONIC LIVER DISEASES | K767 | Hepatorenal syndrome | 572.4 | Hepatorenal syndrome |
| CHRONIC LIVER DISEASES | K778 | Liver disorders in other diseases classified elsewhere |  |  |
| CHRONIC LIVER DISEASES | Q446 | Cystic disease of liver | 751.62 | Congenital cystic disease of liver |
| CHRONIC LIVER DISEASES | Z944 | Liver transplant status | V42.7 | Liver transplant status |
| CHRONIC LIVER DISEASES | K700 | Alcoholic fatty liver | 571.0 | Alcoholic fatty liver |
| CHRONIC LIVER DISEASES | K701 | Alcoholic hepatitis |  |  |
| METABOLIC | E78 | Disorders of lipoprotein metabolism and other lipidaemias |  |  |
| METABOLIC | E20 | Hypoparathyroidism |  |  |
| METABOLIC | E21 | Hyperparathyroidism and other disorders of parathyroid gland |  |  |
| METABOLIC | E22 | Hyperfunction of pituitary gland |  |  |
| METABOLIC | E23 | Hypofunction and other disorders of pituitary gland |  |  |
| METABOLIC | E24 | Cushing syndrome |  |  |
| METABOLIC | E25 | Adrenogenital disorders |  |  |
| METABOLIC | E26 | Hyperaldosteronism |  |  |
| METABOLIC | E27 | Other disorders of adrenal gland |  |  |
| METABOLIC | E28 | Ovarian dysfunction |  |  |
| METABOLIC | E29 | Testicular dysfunction |  |  |
| METABOLIC | E31 | Polyglandular dysfunction |  |  |
| METABOLIC | E34 | Other endocrine disorders |  |  |
| METABOLIC | E35 | Disorders of endocrine glands in diseases classified elsewhere | 246.8 | Other specified disorders of thyroid |
| METABOLIC | E35 | Disorders of endocrine glands in diseases classified elsewhere | 255.8 | Other specified disorders of adrenal glands |
| METABOLIC | E35 | Disorders of endocrine glands in diseases classified elsewhere | 259.8 | Other specified endocrine disorders |
| METABOLIC | E40 | Kwashiorkor | 260 | Kwashiorkor |
| METABOLIC | E41 | Nutritional marasmus | 261 | Nutritional marasmus |
| METABOLIC | E42 | Marasmic kwashiorkor | 260 | Kwashiorkor |
| METABOLIC | E43 | Unspecified severe protein-energy malnutrition | 262 | Other severe protein-calorie malnutrition |
| METABOLIC | E44 | Protein-energy malnutrition of moderate and mild degree |  |  |
| METABOLIC | E45 | Retarded development following protein-energy malnutrition | 263.2 | Arrested development following protein-calorie malnutrition |
| METABOLIC | E46 | Unspecified protein-energy malnutrition | 263.8 | Unspecified protein-calorie malnutrition |
| METABOLIC | E46 | Unspecified protein-energy malnutrition | 263.9 | Unspecified protein-calorie malnutrition |
| METABOLIC | E64 | Sequelae of malnutrition and other nutritional deficiencies |  |  |
| METABOLIC | E70 | Disorders of aromatic amino-acid metabolism |  |  |
| METABOLIC | E71 | Disorders of branched-chain amino-acid metabolism and fatty-acid metabolism |  |  |
| METABOLIC | E72 | Other disorders of amino-acid metabolism |  |  |
| METABOLIC | E74 | Other disorders of carbohydrate metabolism |  |  |
| METABOLIC | E75 | Disorders of sphingolipid metabolism and other lipid storage disorders |  |  |
| METABOLIC | E76 | Disorders of glycosaminoglycan metabolism |  |  |
| METABOLIC | E77 | Disorders of glycoprotein metabolism |  |  |
| METABOLIC | E79 | Disorders of purine and pyrimidine metabolism |  |  |
| METABOLIC | E80 | Disorders of porphyrin and bilirubin metabolism |  |  |
| METABOLIC | E83 | Disorders of mineral metabolism |  |  |
| METABOLIC | E84 | Cystic fibrosis |  |  |
| METABOLIC | E85 | Amyloidosis |  |  |
| METABOLIC | E88 | Other metabolic disorders |  |  |
| METABOLIC | E89 | Postprocedural endocrine and metabolic disorders, not elsewhere classified |  |  |
| METABOLIC | E891 | Postprocedural hypoinsulinaemia | 251.3 | Postsurgical hypoinsulinemia |
| METABOLIC | K903 | Pancreatic steatorrhoea | 579.4 | Pancreatic steatorrhea |
| METABOLIC | K904 | Malabsorption due to intolerance, not elsewhere classified |  |  |
| METABOLIC | K908 | Other intestinal malabsorption |  |  |
| METABOLIC | K909 | Intestinal malabsorption, unspecified | 579.9 | Unspecified intestinal malabsorption |
| METABOLIC | K912 | Postsurgical malabsorption, not elsewhere classified | 579.3 | Postsurgical malabsorption, not elsewhere classified |
| METABOLIC | M83 | Adult osteomalacia |  |  |
| METABOLIC | M88 | Paget disease of bone [osteitis deformans] |  |  |
| METABOLIC | N25 | Disorders resulting from impaired renal tubular function |  |  |
| DIABETES | E10 | Insulin-dependent diabetes mellitus | 250.XX | Diabetes |
| DIABETES | E11 | Non-insulin-dependent diabetes mellitus |  |  |
| DIABETES | E13 | Other specified diabetes mellitus |  |  |
| DIABETES | E14 | Unspecified diabetes mellitus |  |  |
| OBERSITY | E66 | Obesity |  |  |
| NEUROPSYCHIATRIC | F00 | Dementia in Alzheimer disease |  |  |
| NEUROPSYCHIATRIC | F01 | Vascular dementia |  |  |
| NEUROPSYCHIATRIC | F02 | Dementia in other diseases classified elsewhere |  |  |
| NEUROPSYCHIATRIC | F03 | Unspecified dementia |  |  |
| NEUROPSYCHIATRIC | F051 | Delirium superimposed on dementia |  |  |
| NEUROPSYCHIATRIC | G30 | Alzheimer disease |  |  |
| NEUROPSYCHIATRIC | G31 | Other degenerative diseases of nervous system, not elsewhere classified |  |  |
| NEUROPSYCHIATRIC | F30 | Manic episode |  |  |
| NEUROPSYCHIATRIC | F31 | Bipolar affective disorder |  |  |
| NEUROPSYCHIATRIC | F32 | Depressive episode |  |  |
| NEUROPSYCHIATRIC | F33 | Recurrent depressive disorder |  |  |
| NEUROPSYCHIATRIC | F34 | Persistent mood [affective] disorders |  |  |
| NEUROPSYCHIATRIC | F38 | Other mood [affective] disorders |  |  |
| NEUROPSYCHIATRIC | F39 | Unspecified mood [affective] disorder | 296.90 | Unspecified episodic mood disorder |
| NEUROPSYCHIATRIC | F412 | Mixed anxiety and depressive disorder |  |  |
| NEUROPSYCHIATRIC | G40 | Epilepsy |  |  |
| NEUROPSYCHIATRIC | B900 | Sequelae of central nervous system tuberculosis | 137.1 | Late effects of central nervous system tuberculosis |
| NEUROPSYCHIATRIC | D482 | Neoplasm of uncertain or unknown behaviour: Peripheral nerves and autonomic nervous system | 238.1 | Neoplasm of uncertain behavior of connective and other soft tissue |
| NEUROPSYCHIATRIC | G041 | Tropical spastic paraplegia | 344.1 | Paraplegia |
| NEUROPSYCHIATRIC | G09 | Sequelae of inflammatory diseases of central nervous system | 326 | Late effects of intracranial abscess or pyogenic infection |
| NEUROPSYCHIATRIC | G10 | Huntington disease | 333.4 | Huntington's chorea |
| NEUROPSYCHIATRIC | G11 | Hereditary ataxia |  |  |
| NEUROPSYCHIATRIC | G12 | Spinal muscular atrophy and related syndromes |  |  |
| NEUROPSYCHIATRIC | G13 | Systemic atrophies primarily affecting central nervous system in diseases classified elsewhere |  |  |
| NEUROPSYCHIATRIC | G24 | Dystonia |  |  |
| NEUROPSYCHIATRIC | G25 | Other extrapyramidal and movement disorders |  |  |
| NEUROPSYCHIATRIC | G26 | Extrapyramidal and movement disorders in diseases classified elsewhere | 333.99 | Other extrapyramidal diseases and abnormal movement disorders |
| NEUROPSYCHIATRIC | G32 | Other degenerative disorders of nervous system in diseases classified elsewhere |  |  |
| NEUROPSYCHIATRIC | G37 | Other demyelinating diseases of central nervous system |  |  |
| NEUROPSYCHIATRIC | G51 | Facial nerve disorders |  |  |
| NEUROPSYCHIATRIC | G52 | Disorders of other cranial nerves |  |  |
| NEUROPSYCHIATRIC | G53 | Cranial nerve disorders in diseases classified elsewhere | 352.9 | Unspecified disorder of cranial nerves |
| NEUROPSYCHIATRIC | G70 | Myasthenia gravis and other myoneural disorders |  |  |
| NEUROPSYCHIATRIC | G71 | Primary disorders of muscles |  |  |
| NEUROPSYCHIATRIC | G723 | Periodic paralysis | 359.3 | Periodic paralysis |
| NEUROPSYCHIATRIC | G724 | Inflammatory myopathy, not elsewhere classified |  |  |
| NEUROPSYCHIATRIC | G728 | Other specified myopathies |  |  |
| NEUROPSYCHIATRIC | G729 | Myopathy, unspecified | 359.9 | Myopathy, unspecified |
| NEUROPSYCHIATRIC | G73 | Disorders of myoneural junction and muscle in diseases classified elsewhere |  |  |
| NEUROPSYCHIATRIC | G80 | Cerebral palsy |  |  |
| NEUROPSYCHIATRIC | G81 | Hemiplegia |  |  |
| NEUROPSYCHIATRIC | G82 | Paraplegia and tetraplegia |  |  |
| NEUROPSYCHIATRIC | G83 | Other paralytic syndromes |  |  |
| NEUROPSYCHIATRIC | G90 | Disorders of autonomic nervous system |  |  |
| NEUROPSYCHIATRIC | G91 | Hydrocephalus |  |  |
| NEUROPSYCHIATRIC | G938 | Other specified disorders of brain |  |  |
| NEUROPSYCHIATRIC | G939 | Disorder of brain, unspecified | 348.9 | Unspecified condition of brain |
| NEUROPSYCHIATRIC | G95 | Other diseases of spinal cord |  |  |
| NEUROPSYCHIATRIC | G99 | Other disorders of nervous system in diseases classified elsewhere |  |  |
| NEUROPSYCHIATRIC | M471 | Other spondylosis with myelopathy |  |  |
| NEUROPSYCHIATRIC | Q00 | Anencephaly and similar malformations |  |  |
| NEUROPSYCHIATRIC | Q01 | Encephalocele |  |  |
| NEUROPSYCHIATRIC | Q02 | Microcephaly | 742.1 | Microcephalus |
| NEUROPSYCHIATRIC | Q03 | Congenital hydrocephalus |  |  |
| NEUROPSYCHIATRIC | Q04 | Other congenital malformations of brain |  |  |
| NEUROPSYCHIATRIC | Q05 | Spina bifida |  |  |
| NEUROPSYCHIATRIC | Q06 | Other congenital malformations of spinal cord |  |  |
| NEUROPSYCHIATRIC | Q07 | Other congenital malformations of nervous system |  |  |
| NEUROPSYCHIATRIC | Q760 | Spina bifida occulta | 756.17 | Spina bifida occulta |
| NEUROPSYCHIATRIC | F04 | Organic amnesic syndrome, not induced by alcohol and other psychoactive substances | 294.0 | Amnestic disorder in conditions classified elsewhere |
| NEUROPSYCHIATRIC | F06 | Other mental disorders due to brain damage and dysfunction and to physical disease |  |  |
| NEUROPSYCHIATRIC | F07 | Personality and behavioural disorders due to brain disease, damage and dysfunction |  |  |
| NEUROPSYCHIATRIC | F09 | Unspecified organic or symptomatic mental disorder | 310.9 | Unspecified nonpsychotic mental disorder following organic brain damage |
| NEUROPSYCHIATRIC | F102 | Mental and behavioural disorders due to use of alcohol: Dependence syndrome |  |  |
| NEUROPSYCHIATRIC | F106 | Mental and behavioural disorders due to use of alcohol: Amnesic syndrome |  |  |
| NEUROPSYCHIATRIC | F107 | Mental and behavioural disorders due to use of alcohol: Residual and late-onset psychotic disorder |  |  |
| NEUROPSYCHIATRIC | F112 | Mental and behavioural disorders due to use of opioids: Dependence syndrome |  |  |
| NEUROPSYCHIATRIC | F116 | Mental and behavioural disorders due to use of opioids: Amnesic syndrome |  |  |
| NEUROPSYCHIATRIC | F117 | Mental and behavioural disorders due to use of opioids: Residual and late-onset psychotic disorder |  |  |
| NEUROPSYCHIATRIC | F122 | Mental and behavioural disorders due to use of cannabinoids: Dependence syndrome |  |  |
| NEUROPSYCHIATRIC | F126 | Mental and behavioural disorders due to use of cannabinoids: Amnesic syndrome |  |  |
| NEUROPSYCHIATRIC | F127 | Mental and behavioural disorders due to use of cannabinoids: Residual and late-onset psychotic disorder |  |  |
| NEUROPSYCHIATRIC | F132 | Mental and behavioural disorders due to use of sedatives or hypnotics: Dependence syndrome |  |  |
| NEUROPSYCHIATRIC | F136 | Mental and behavioural disorders due to use of sedatives or hypnotics: Amnesic syndrome |  |  |
| NEUROPSYCHIATRIC | F137 | Mental and behavioural disorders due to use of sedatives or hypnotics: Residual and late-onset psychotic disorder |  |  |
| NEUROPSYCHIATRIC | F142 | Mental and behavioural disorders due to use of cocaine: Dependence syndrome |  |  |
| NEUROPSYCHIATRIC | F146 | Mental and behavioural disorders due to use of cocaine: Amnesic syndrome |  |  |
| NEUROPSYCHIATRIC | F147 | Mental and behavioural disorders due to use of cocaine: Residual and late-onset psychotic disorder |  |  |
| NEUROPSYCHIATRIC | F152 | Mental and behavioural disorders due to use of other stimulants, including caffeine: Dependence syndrome |  |  |
| NEUROPSYCHIATRIC | F156 | Mental and behavioural disorders due to use of other stimulants, including caffeine: Amnesic syndrome |  |  |
| NEUROPSYCHIATRIC | F157 | Mental and behavioural disorders due to use of other stimulants, including caffeine: Residual and late-onset psychotic disorder |  |  |
| NEUROPSYCHIATRIC | F162 | Mental and behavioural disorders due to use of hallucinogens: Dependence syndrome |  |  |
| NEUROPSYCHIATRIC | F166 | Mental and behavioural disorders due to use of hallucinogens: Amnesic syndrome |  |  |
| NEUROPSYCHIATRIC | F167 | Mental and behavioural disorders due to use of hallucinogens: Residual and late-onset psychotic disorder |  |  |
| NEUROPSYCHIATRIC | F172 | Mental and behavioural disorders due to use of tobacco: Dependence syndrome |  |  |
| NEUROPSYCHIATRIC | F176 | Mental and behavioural disorders due to use of tobacco: Amnesic syndrome |  |  |
| NEUROPSYCHIATRIC | F177 | Mental and behavioural disorders due to use of tobacco: Residual and late-onset psychotic disorder |  |  |
| NEUROPSYCHIATRIC | F182 | Mental and behavioural disorders due to use of volatile solvents: Dependence syndrome |  |  |
| NEUROPSYCHIATRIC | F186 | Mental and behavioural disorders due to use of volatile solvents: Amnesic syndrome |  |  |
| NEUROPSYCHIATRIC | F187 | Mental and behavioural disorders due to use of volatile solvents: Residual and late-onset psychotic disorder |  |  |
| NEUROPSYCHIATRIC | F192 | Mental and behavioural disorders due to multiple drug use and use of other psychoactive substances: Dependence syndrome |  |  |
| NEUROPSYCHIATRIC | F196 | Mental and behavioural disorders due to multiple drug use and use of other psychoactive substances: Amnesic syndrome |  |  |
| NEUROPSYCHIATRIC | F197 | Mental and behavioural disorders due to multiple drug use and use of other psychoactive substances: Residual and late-onset psychotic disorder |  |  |
| NEUROPSYCHIATRIC | F50 | Eating disorders |  |  |
| NEUROPSYCHIATRIC | F52 | Sexual dysfunction, not caused by organic disorder or disease |  |  |
| NEUROPSYCHIATRIC | F60 | Specific personality disorders |  |  |
| NEUROPSYCHIATRIC | F61 | Mixed and other personality disorders |  |  |
| NEUROPSYCHIATRIC | F62 | Enduring personality changes, not attributable to brain damage and disease |  |  |
| NEUROPSYCHIATRIC | F63 | Habit and impulse disorders |  |  |
| NEUROPSYCHIATRIC | F68 | Other disorders of adult personality and behaviour |  |  |
| NEUROPSYCHIATRIC | F70 | Mild mental retardation | 317 | Mild intellectual disabilities |
| NEUROPSYCHIATRIC | F71 | Moderate mental retardation | 318.0 | Moderate intellectual disabilities |
| NEUROPSYCHIATRIC | F72 | Severe mental retardation | 318.1 | Severe intellectual disabilities |
| NEUROPSYCHIATRIC | F73 | Profound mental retardation | 318.2 | Profound intellectual disabilities |
| NEUROPSYCHIATRIC | F78 | Other mental retardation | 319 | Unspecified intellectual disabilities |
| NEUROPSYCHIATRIC | F79 | Unspecified mental retardation | 319 | Unspecified intellectual disabilities |
| NEUROPSYCHIATRIC | F80 | Specific developmental disorders of speech and language |  |  |
| NEUROPSYCHIATRIC | F81 | Specific developmental disorders of scholastic skills |  |  |
| NEUROPSYCHIATRIC | F82 | Specific developmental disorder of motor function | 315.4 | Developmental coordination disorder |
| NEUROPSYCHIATRIC | F83 | Mixed specific developmental disorders |  |  |
| NEUROPSYCHIATRIC | F84 | Pervasive developmental disorders |  |  |
| NEUROPSYCHIATRIC | F88 | Other disorders of psychological development | 315.8 | Other specified delays in development |
| NEUROPSYCHIATRIC | F89 | Unspecified disorder of psychological development | 315.9 | Unspecified delay in development |
| NEUROPSYCHIATRIC | F95 | Tic disorders |  |  |
| NEUROPSYCHIATRIC | F99 | Mental disorder, not otherwise specified | 300.9 | Unspecified nonpsychotic mental disorder |
| NEUROPSYCHIATRIC | G20 | Parkinson disease | 332.0 | Paralysis agitans |
| NEUROPSYCHIATRIC | G21 | Secondary parkinsonism |  |  |
| NEUROPSYCHIATRIC | G22 | Parkinsonism in diseases classified elsewhere |  |  |
| NEUROPSYCHIATRIC | G23 | Other degenerative diseases of basal ganglia |  |  |
| NEUROPSYCHIATRIC | F20 | Schizophrenia |  |  |
| NEUROPSYCHIATRIC | F22 | Persistent delusional disorders | 297.0 | Paranoid state, simple |
| NEUROPSYCHIATRIC | F22 | Persistent delusional disorders | 297.1 | Delusional disorder |
| NEUROPSYCHIATRIC | F22 | Persistent delusional disorders | 297.2 | Paraphrenia |
| NEUROPSYCHIATRIC | F24 | Induced delusional disorder | 297.3 | Shared psychotic disorder |
| NEUROPSYCHIATRIC | F25 | Schizoaffective disorders |  |  |
| NEUROPSYCHIATRIC | F28 | Other nonorganic psychotic disorders | 298.9 | Unspecified psychosis |
| RESPIRATORY | J45 | Asthma |  |  |
| RESPIRATORY | J41 | Simple and mucopurulent chronic bronchitis |  |  |
| RESPIRATORY | J42 | Unspecified chronic bronchitis | 491.9 | Unspecified chronic bronchitis |
| RESPIRATORY | J43 | Emphysema |  |  |
| RESPIRATORY | J44 | Other chronic obstructive pulmonary disease |  |  |
| RESPIRATORY | J47 | Bronchiectasis |  |  |
| RESPIRATORY | B909 | Sequelae of respiratory and unspecified tuberculosis | 137.0 | Late effects of respiratory or unspecified tuberculosis |
| RESPIRATORY | E662 | Extreme obesity with alveolar hypoventilation | 278.03 | Obesity hypoventilation syndrome |
| RESPIRATORY | J60 | Coalworker pneumoconiosis | 500 | Coal workers' pneumoconiosis |
| RESPIRATORY | J61 | Pneumoconiosis due to asbestos and other mineral fibres | 501 | Asbestosis |
| RESPIRATORY | J62 | Pneumoconiosis due to dust containing silica |  |  |
| RESPIRATORY | J63 | Pneumoconiosis due to other inorganic dusts |  |  |
| RESPIRATORY | J64 | Unspecified pneumoconiosis | 505 | Pneumoconiosis, unspecified |
| RESPIRATORY | J65 | Pneumoconiosis associated with tuberculosis | 505 | Pneumoconiosis, unspecified |
| RESPIRATORY | J66 | Airway disease due to specific organic dust |  |  |
| RESPIRATORY | J67 | Hypersensitivity pneumonitis due to organic dust |  |  |
| RESPIRATORY | J684 | Chronic respiratory conditions due to chemicals, gases, fumes and vapours | 506.4 | Chronic respiratory conditions due to fumes and vapors |
| RESPIRATORY | J701 | Chronic and other pulmonary manifestations due to radiation | 508.1 | Chronic and other pulmonary manifestations due to radiation |
| RESPIRATORY | J703 | Chronic drug-induced interstitial lung disorders | 508.8 | Respiratory conditions due to other specified external agents |
| RESPIRATORY | J704 | Drug-induced interstitial lung disorders, unspecified | 508.8 | Respiratory conditions due to other specified external agents |
| RESPIRATORY | J84 | Other interstitial pulmonary diseases |  |  |
| RESPIRATORY | J92 | Pleural plaque |  |  |
| RESPIRATORY | J941 | Fibrothorax | 511.0 | Pleurisy without mention of effusion or current tuberculosis |
| RESPIRATORY | J953 | Chronic pulmonary insufficiency following surgery | 518.52 | Other pulmonary insufficiency, not elsewhere classified, following trauma and surgery |
| RESPIRATORY | J955 | Postprocedural subglottic stenosis | 997.39 | Other respiratory complications |
| RESPIRATORY | J961 | Chronic respiratory failure |  |  |
| RESPIRATORY | J98 | Other respiratory disorders |  |  |
| RESPIRATORY | Q33 | Congenital malformations of lung |  |  |
| RESPIRATORY | Q34 | Other congenital malformations of respiratory system |  |  |
| RESPIRATORY | Z902 | Acquired absence of lung [part of] | V45.76 | Acquired absence of organ, lung |
| RESPIRATORY | Z942 | Lung transplant status | V42.6 | Postsurgical states following surgery of eye and adnexa |
| RESPIRATORY | Z943 | Heart and lungs transplant status | V42.1 | Postsurgical renal dialysis status |
| RESPIRATORY | Z943 | Heart and lungs transplant status | V42.6 | Postsurgical states following surgery of eye and adnexa |
| RESPIRATORY | Z963 | Presence of artificial larynx | V43.81 | Larynx replacement |

**Table A2:** **STROBE Statement—checklist of items that should be included in reports of observational studies**

|  | **Item No.** | **Recommendation** | **Page  No.** | **Relevant text from manuscript** |
| --- | --- | --- | --- | --- |
| **Title and abstract** | 1 | (*a*) Indicate the study’s design with a commonly used term in the title or the abstract | 1,3 | Title: Disparities in COVID-19 Clinical Outcomes and Vaccination Coverage Among Migrants with HIV in the PISCIS Cohort: A Population-Based Propensity Score-Matched Analysis.  Abstract methods: Using data from the PISCIS cohort of people with HIV (PWH) in Catalonia, Spain, we investigated COVID-19 outcomes and vaccination coverage. |
|  |  | (*b*) Provide in the abstract an informative and balanced summary of what was done and what was found | 3 | Among 10,640 PWH we compared migrants and non-migrants assessing rates of SARS-CoV-2 testing, diagnosis and associated clinical outcomes through propensity-score matching and multivariable Cox regression. Migrants with HIV (MWH) had fewer SARS-CoV-2 tests (67.8% vs. 72.1%, p<0.0001) but similar COVID-19 diagnoses (29.2% vs. 29.4%, p=0.847) compared to Spanish natives. Migrants had lower complete vaccination (78.9% vs. 85.1%, p<0.0001) and booster doses (63.0% vs. 65.5%, p=0.027). COVID-19 hospitalizations (8.1% vs. 5.1%, p<0.0001) and ICU admissions (2.9% vs. 1.2%, p<0.0001) were higher among migrants, with similar hospitalization duration (5.5 vs. 4.0 days, p=0.098) and mortality (3 [0.2%] vs. 6 [0.4%], p=0.510). Age ≥40 years, CD4 counts <200 cells/μL, ≥2 comorbidities, and incomplete/non-reception of the SARS-CoV-2 vaccine increased the risk of severe COVID-19 among migrants. |
| **Introduction** | | | |  |
| Background/rationale | 2 | Explain the scientific background and rationale for the investigation being reported | 5-6 | Introduction |
| Objectives | 3 | State specific objectives, including any prespecified hypotheses | 6 | In this study, we leveraged a large population-based cohort of PLHIV in Catalonia, Spain, to compare SARS-CoV-2 testing, diagnosis, hospitalisation, intensive care unit (ICU) admission, hospitalisation length, mortality, and vaccination coverage between MWH and a well-matched native group. Additionally, we identified the factors associated with severe COVID-19 and vaccination coverage in the migrant group. |
| **Methods** | | | |  |
| Study design | 4 | Present key elements of study design early in the paper | 7 | We conducted a retrospective cohort study using data from the PISCIS cohort of PLHIV in Catalonia, Spain. |
| Setting | 5 | Describe the setting, locations, and relevant dates, including periods of recruitment, exposure, follow-up, and data collection | 7 |  |
| Participants | 6 | (*a*) *Cohort study*—Give the eligibility criteria, and the sources and methods of selection of participants. Describe methods of follow-up  *Case-control study*—Give the eligibility criteria, and the sources and methods of case ascertainment and control selection. Give the rationale for the choice of cases and controls  *Cross-sectional study*—Give the eligibility criteria, and the sources and methods of selection of participants | 7 | We excluded patients who were reported as deceased before March 1, 2020, as well as those who had no information regarding their country of origin. For the analysis of vaccine coverage, we further excluded subjects who were reported as deceased before December 27, 2020, and individuals who had not utilized any health services in the prior 12 months to minimize the underestimation of vaccine uptake. The eligible participants were divided into two groups based on their country of origin: Spanish and migrants. |
|  |  | (*b*) *Cohort study*—For matched studies, give matching criteria and number of exposed and unexposed  *Case-control study*—For matched studies, give matching criteria and the number of controls per case |  |  |
| Variables | 7 | Clearly define all outcomes, exposures, predictors, potential confounders, and effect modifiers. Give diagnostic criteria, if applicable | 8 |  |
| Data sources/ measurement | 8* | For each variable of interest, give sources of data and details of methods of assessment (measurement). Describe comparability of assessment methods if there is more than one group | 8 |  |
| Bias | 9 | Describe any efforts to address potential sources of bias | 7 |  |
| Study size | 10 | Explain how the study size was arrived at | 7 |  |

Continued on next page

| Quantitative variables | 11 | Explain how quantitative variables were handled in the analyses. If applicable, describe which groupings were chosen and why | 9 |  |
| --- | --- | --- | --- | --- |
| Statistical methods | 12 | (*a*) Describe all statistical methods, including those used to control for confounding | 9 |  |
|  |  | (*b*) Describe any methods used to examine subgroups and interactions | 9 |  |
|  |  | (*c*) Explain how missing data were addressed | 7 |  |
|  |  | (*d*) *Cohort study*—If applicable, explain how loss to follow-up was addressed  *Case-control study*—If applicable, explain how matching of cases and controls was addressed  *Cross-sectional study*—If applicable, describe analytical methods taking account of sampling strategy | 7 |  |
|  |  | (*e*) Describe any sensitivity analyses | Supplementary materials |  |
| **Results** | | | | |
| Participants | 13* | (a) Report numbers of individuals at each stage of study—eg numbers potentially eligible, examined for eligibility, confirmed eligible, included in the study, completing follow-up, and analysed | 11,12 |  |
|  |  | (b) Give reasons for non-participation at each stage |  |  |
|  |  | (c) Consider use of a flow diagram |  |  |
| Descriptive data | 14* | (a) Give characteristics of study participants (eg demographic, clinical, social) and information on exposures and potential confounders | 11,12 |  |
|  |  | (b) Indicate number of participants with missing data for each variable of interest | 26-28 |  |
|  |  | (c) *Cohort study*—Summarise follow-up time (eg, average and total amount) | 26-28 |  |
| Outcome data | 15* | *Cohort study*—Report numbers of outcome events or summary measures over time | 26-28 |  |
|  |  | *Case-control study—*Report numbers in each exposure category, or summary measures of exposure |  |  |
|  |  | *Cross-sectional study—*Report numbers of outcome events or summary measures |  |  |
| Main results | 16 | (*a*) Give unadjusted estimates and, if applicable, confounder-adjusted estimates and their precision (eg, 95% confidence interval). Make clear which confounders were adjusted for and why they were included | 29-31 |  |
|  |  | (*b*) Report category boundaries when continuous variables were categorized |  |  |
|  |  | (*c*) If relevant, consider translating estimates of relative risk into absolute risk for a meaningful time period |  |  |

Continued on next page

| Other analyses | 17 | Report other analyses done—eg analyses of subgroups and interactions, and sensitivity analyses | Supplementary materials |  |
| --- | --- | --- | --- | --- |
| **Discussion** | | | | |
| Key results | 18 | Summarise key results with reference to study objectives | 13 | In this propensity score-matched cohort of PLHIV in Catalonia, Spain, we found that MLHIV in Catalonia had higher rates of COVID-19-associated hospitalisations and ICU admissions compared to their Spanish counterparts. Additionally, migrants had lower rates of SARS-CoV-2 testing and vaccination coverage, although the rates of SARS-CoV-2 diagnosis were similar between the two groups. Migrants who were 40 years old or older, had a CD4 count of less than 200 cells per μL, had two or more comorbidities, and those who were unvaccinated or incompletely vaccinated against SARS-CoV-2 were found to have a significantly increased risk of severe COVID-19. |
| Limitations | 19 | Discuss limitations of the study, taking into account sources of potential bias or imprecision. Discuss both direction and magnitude of any potential bias | 16-17 |  |
| Interpretation | 20 | Give a cautious overall interpretation of results considering objectives, limitations, multiplicity of analyses, results from similar studies, and other relevant evidence | 13-17 |  |
| Generalisability | 21 | Discuss the generalisability (external validity) of the study results | 13-17 |  |
| **Other information** | |  | | |
| Funding | 22 | Give the source of funding and the role of the funders for the present study and, if applicable, for the original study on which the present article is based | 19 |  |
